# THMC Modeling for CO_2_ Geological Storage: Advances, Challenges, and Prospects

**DOI:** 10.1021/acsomega.6c00226

**Published:** 2026-03-05

**Authors:** Jia Chang, Keyao Lin, Ning Wei, Shengnan Liu, Meng Jing, Chenlong Yang, Tianyu Liu

**Affiliations:** † CHN Energy Yulin Chemical Co., Ltd., Yulin 719302, China; ‡ 74613 Institute of Rock and Soil Mechanics, Chinese Academy of Sciences, Wuhan 430071, China; § University of Chinese Academy of Sciences, Beijing 100049, China

## Abstract

CO_2_ geological storage (CGS) is an important way to reach carbon neutrality. Its long-term safety and effectiveness depend on the interplay of thermal, hydraulic, mechanical, and chemical (THMC) processes. However, contemporary research frequently examines discrete processes or particular geological contexts, resulting in a fragmented understanding of THMC interactions and the absence of a unified framework for multiphysics simulation. This paper presents the first comprehensive review of the application landscape of THMC coupling in CGS. It elucidates the fundamental processes of each physical field and their interacting mechanisms, spanning from two- to four-field coupling. It also looks at the pros and cons of common numerical tactics, computational methodologies, and software platforms, as well as how useful they are. It looks at the most important uses of THMC models in site screening, wellbore integrity evaluation, and storage evolution forecasts using common situations such as deep saline aquifers and depleted oil and gas reservoirs. This paper talks about current problems, such as computing efficiency, parameter uncertainty, and lack of validation. It does this by suggesting eight development ideas, such as using intelligent algorithms, multiscale modeling, data assimilation, and standardized platforms. This evaluation encourages a better understanding of how things work, better engineering techniques, and the growth of CGS into a safer, more efficient, and larger-scale application.

## Graphical abstract

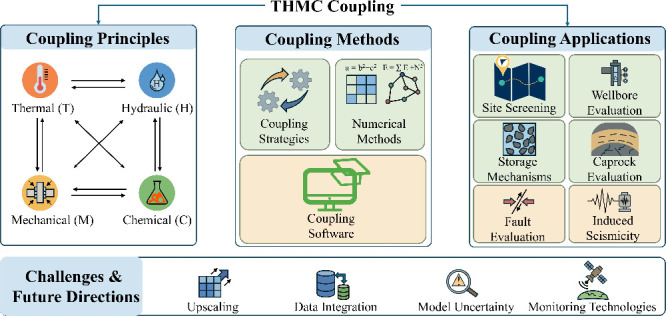

## Highlights

1. First systematic review of THMC coupling application landscape in CO_2_ geological storage.
2. Evaluates mainstream numerical strategies, methods, and software for THMC simulation.
3. Analyzes key applications in site screening, wellbore integrity, and storage evolution.
4. Proposes eight forward-looking recommendations for THMC modeling development.
5. Supports mechanistic insight and engineering practice for safer, large-scale CO_2_ storage.

## 1 Introduction

Since the Industrial Revolution, human activities have led to a sharp increase in the concentration of greenhouse gases (especially CO_2_), making it a primary driver of global warming.^1^ Assessments by the Intergovernmental Panel on Climate Change (IPCC) indicate that the global average surface temperature from 2011–2020 was approximately 1.1 °C higher than the preindustrial level (1850–1900), triggering a series of severe ecological and environmental crises.^2^ Including accelerated polar ice sheet melting, sea-level rise threatening coastal and island security, significant increases in the frequency and intensity of extreme weather events (such as intense tropical cyclones, heavy rainfall, and persistent droughts), and serious threats to global food production, water resource distribution, and biodiversity.^3^ To address this challenge, there is a global consensus on the need for urgent and robust action to achieve carbon neutrality.^4,5^ Among various emission reduction pathways, carbon capture, utilization, and storage (CCUS) technology is widely regarded as a key solution for achieving low-carbon utilization of fossil energy.^6,7^ Among these, the safe and permanent storage of captured CO_2_ in subsurface geological formations is crucial for preventing its re-entry into the atmosphere.^8,9^ Therefore, achieving large-scale, economically viable, and long-term safe CO_2_ geological storage (CGS) has become a central issue in current scientific research and engineering applications.^10^

CGS technology involves injecting captured CO_2_ into deep saline aquifers, depleted oil and gas reservoirs, or unminable coal seams, utilizing caprock sealing and physicochemical trapping mechanisms to achieve long-term or permanent CO_2_ storage.^11–13^ This technology holds immense potential, with the global geological storage capacity estimated at 1,460 (1290–2710) Gt, sufficient to accommodate several centuries of emissions.^14^ But CO_2_ injection is not just a way to fill something; it is a complicated process that throws off the balance of the subsurface.^15^ The injected CO_2_ interacts with reservoir rocks, pore fluids, and caprocks through multiphase processes, leading to significant changes and reciprocal feedback in the thermal, hydraulic, mechanical, and chemical domains.^16,17^ For example, injection pressure can change the stress in the ground, which can cause microfractures or faults to become active again. CO_2_ reacts with formation water to make carbonic acid, which dissolves minerals and changes the pore structure and changes the flow properties. Changes in temperature can also cause thermal stresses and changes in fluid properties.^18^ These processes are interwoven, forming a typical thermo-hydro-mechanical-chemical (THMC) multifield coupling system.^19,20^ Therefore, a deep understanding and accurate simulation of the THMC coupling processes are crucial for predicting the long-term security and efficiency of a storage complex.

THMC multifield coupling simulation is crucial for the safety assessment, efficiency optimization, and risk management of CGS, demonstrating particularly prominent application value in specific storage scenarios.^21^ As shown in Figure 1, the THMC coupling effects collectively govern the key processes within the storage system. Taking deep saline formation storage as an example, simulations can quantify the impact of injection pressure on caprock integrity, predicting whether it induces fracture propagation or microseismical activity; simultaneously track the migration path of the CO_2_ plume, and, combined with chemical reactions, predict the long-term alteration of reservoir porosity and permeability due to mineral dissolution and precipitation, thereby assessing storage space utilization efficiency and long-term containment security.^22,23^ In depleted oil and gas reservoir storage, besides the aforementioned processes, special consideration must be given to the reservoir pressure depletion and changes in rock mechanical properties resulting from historical production.^24^ The pressure rebound and effective stress changes induced by CO_2_ injection may have unique impacts on wellbore integrity and reservoir skeleton stability.^25^ Furthermore, the thermal stress generated by injecting cooler CO_2_ (relative to the formation temperature), constituting thermo-hydro-mechanical (THM) coupling, is also a key factor in assessing the geomechanical behavior near the wellbore.^26^ Through high-fidelity THMC coupled simulations, injection parameters (such as rate and temperature) can be optimized during the project design phase, key indicators can be monitored in real-time during the operational phase, and long-term evolution can be predicted during the postinjection phase, thereby providing a scientific basis for full-cycle management and ensuring the long-term safety and reliability of the project.^27^

**Figure 1:**
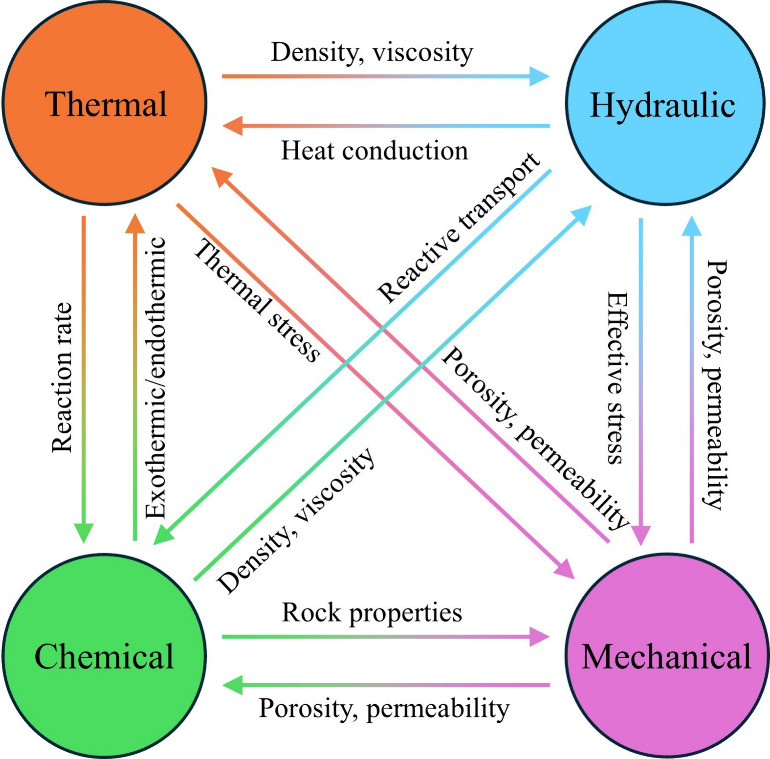
Schematic diagram of THMC coupling mechanisms in CGS.

Although the THMC multifield coupling simulation is crucial for understanding and predicting the behavior of CGS, a systematic review synthesizing the progress in this field is currently lacking. Existing reviews mostly focus on specific aspects. For example, Rathnaweera et al.^28^ summarized THMC coupling processes in enhanced geothermal systems, noting that mechanisms such as pore pressure diffusion, temperature changes, and stress-assisted corrosion collectively influence fault reactivation; Gholami et al.,^29^ by analyzing experimental data from over 50 carbon storage sites worldwide, generalized the interaction mechanisms between CO_2_, rock, and cement, emphasizing that geochemical reactions, temperature–pressure conditions, and cementing quality are primary causes of leakage, and proposed a risk assessment framework for improved safety; As shown in Figure 2, Hosseini et al.^30^ systematically discussed best practices for the dynamic modeling of CO_2_ storage in deep saline aquifers, focusing on the analysis of trapping mechanisms and uncertainties in CO_2_ migration, and proposed interdisciplinary technical countermeasures. However, these reviews are often confined to specific processes (such as mechanical, chemical, or transport) or specific geological settings, failing to systematically integrate and critique the comprehensive application of the four THMC fields and their full coupling mechanisms in CGS, thus making it difficult to establish a unified knowledge framework for guiding multifield coupling simulation practices.

**Figure 2:**
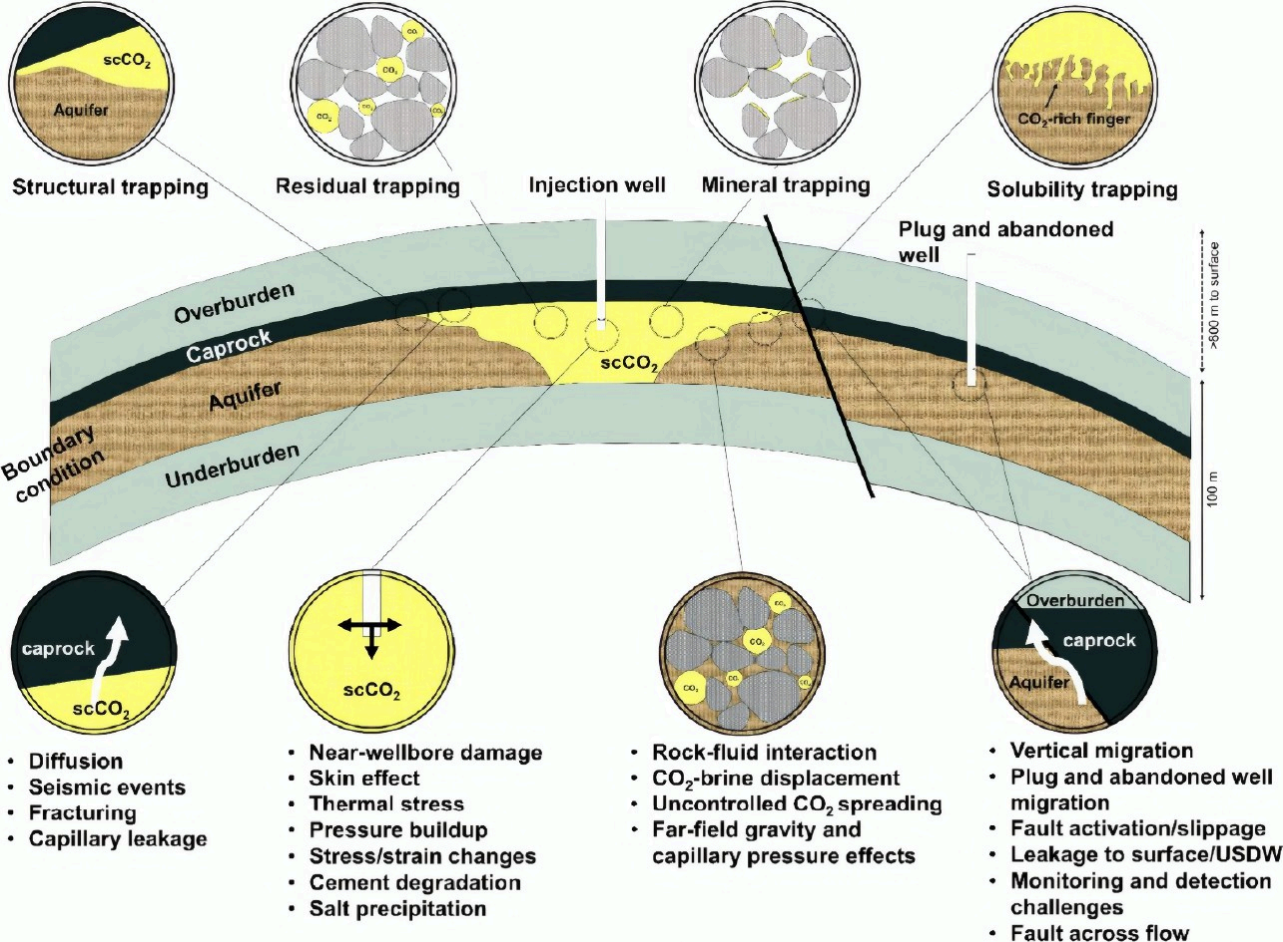
Schematic diagram of uncertainties in the CO_2_ storage and migration in saline aquifers. This figure was reproduced with permission from ref 30. Copyright 2024 Elsevier.

In summary, a deep understanding of THMC multifield coupling processes is the scientific foundation for ensuring the successful application of CGS technology, yet current research still lacks a systematic review on this topic. This study thoroughly assesses and forecasts the implementation of THMC multifield coupling simulation in CGS research. This paper initially explains the main processes of each physical domain (thermal, hydraulic, mechanical, and chemical) in the THMC coupling system and how they work together, as shown in Figure 3. It then analyzes coupling strategies, numerical methodologies, and the features and suitable applications of pertinent coupling software for THMC multifield coupling. By incorporating case studies from standard storage contexts, including deep saline deposits and exhausted hydrocarbon reservoirs, it evaluates the importance of THMC coupling modeling in addressing particular scientific and engineering issues. Finally, it summarizes existing accomplishments, identifies key challenges in current research, and proposes future research directions. This review aims to provide researchers and engineers with a clear knowledge framework, deepen the understanding of multiphysics coupling behavior in CGS, and offer theoretical references for the safe deployment of the technology and engineering practices.

**Figure 3:**
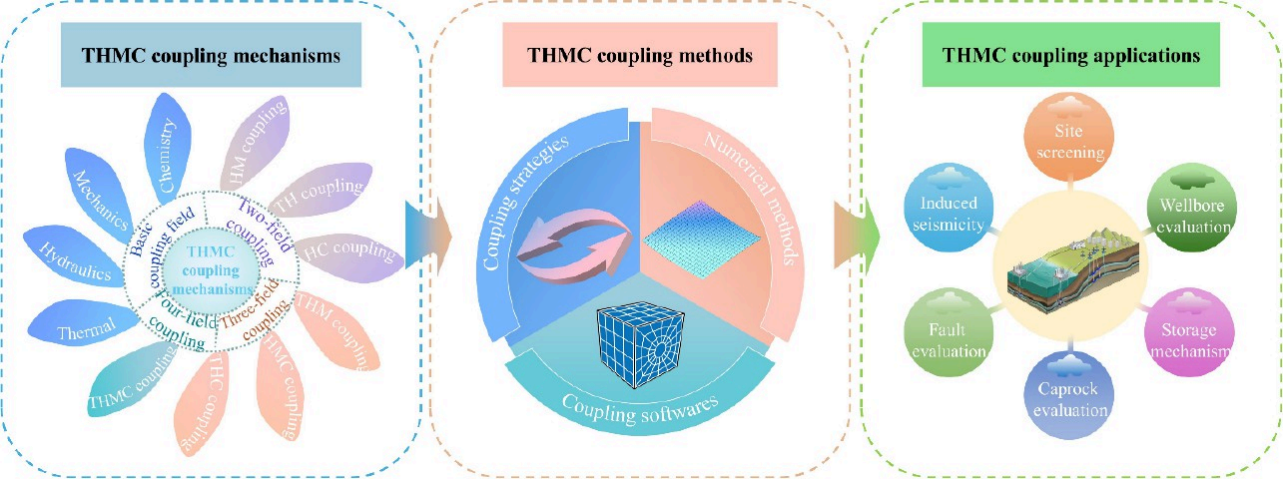
Research flowchart of this study.

## 2 THMC Coupling Mechanisms

### 2.1 Fundamental Coupling Fields

#### 2.1.1 Thermal Process

In the CGS system, the thermal process is a fundamental physical field involving temperature field evolution, heat transport, and associated thermal effects. Typically, a significant temperature difference exists between the injected CO_2_ fluid and the deep reservoir, thereby inducing complex heat exchange processes.^31^ Heat transport is primarily achieved through thermal convection and thermal conduction, with thermal convection accompanying CO_2_ migration being a key mechanism determining the reservoir’s temperature distribution. These processes induce significant thermal effects, most notably the Joule-Thomson (J-T) cooling effect—a rapid temperature drop caused by the throttling and expansion of high-pressure CO_2_ in the wellbore and near-wellbore zone—as well as thermal expansion or cold contraction of reservoir rocks and fluids due to temperature changes.^32,33^ Understanding these fundamental thermal processes is essential for analyzing subsequent thermal-hydraulic (TH) and THM coupling interactions.

Figure 4a shows the relationship between the Joule-Thomson coefficient (μJT) of the CO_2_+CH_3_OH mixture and pressure (0–200 MPa) within the temperature range of 263.15 K-313.15 K, where it is evident that μJT exhibits a significant decreasing trend with increasing pressure, and the peak μJT value at high-temperature conditions (e.g., 313.15 K) is significantly higher than that at low-temperature conditions (e.g., 263.15 K).^34^ Figure 4b presents the distribution of μJT calculated by different thermodynamic models (PR EoS, TraPPE, SAFT-y Mie, EPM2, etc.) under conditions of 300–900 K temperature and 30–100 MPa pressure, wherein the models show that the influence of temperature variation on μJT is more significant in the low to intermediate temperature region (<500 K), while the sensitivity of μJT to temperature regulation is markedly reduced in the high-temperature region (>500 K).^35^

**Figure 4:**
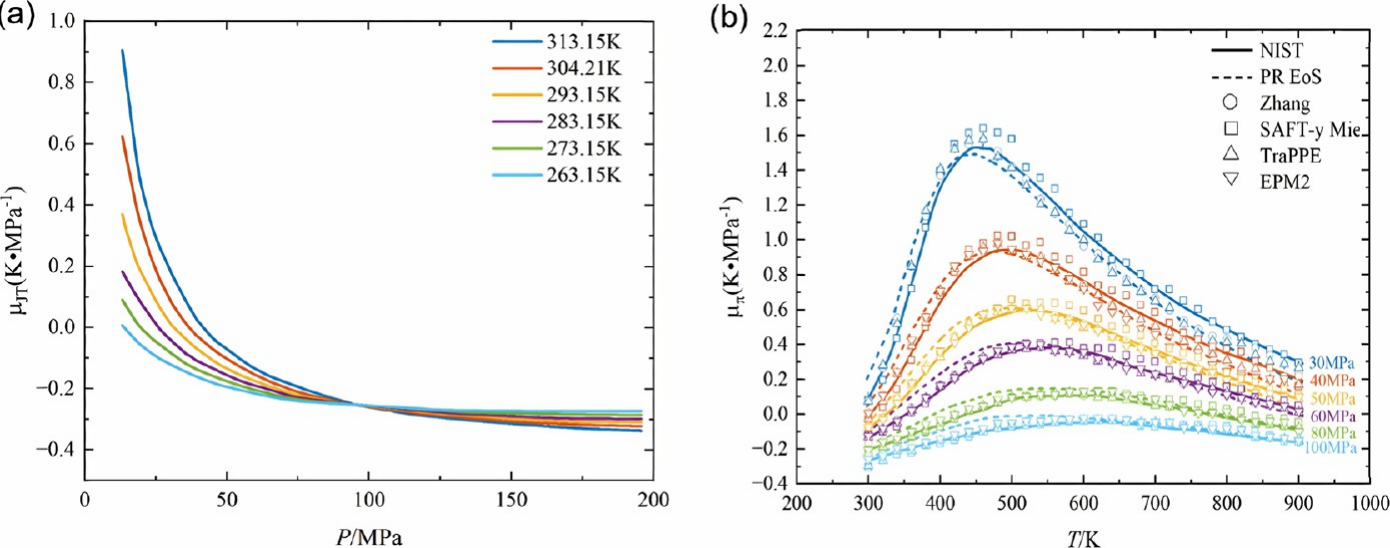
(a) Variation of μJT with pressure for the CO_2_ + CH_3_OH mixture under different temperatures; (b) distribution of μJT of CO_2_ calculated by different thermodynamic models under various temperature and pressure conditions. This figure was reproduced with permission from ref 36. Copyright 2024 Elsevier.

During actual storage operations, thermal effects have a direct impact on the injection safety and storage efficiency. The injection of cool CO_2_ into the near-wellbore area causes a big drop in temperature. This creates a lot of thermal stress, changes the stress state in the area, changes the mechanical properties of the rock and the pressure needed to break it, and may even cause or spread microfractures.^37^ This THM coupling effect is an important part of checking the integrity of the wellbore, the stability of the reservoir, and the sealing ability of the caprock. Moreover, temperature changes also change the physical qualities of CO_2_, such as its density and viscosity, and how it behaves in different phases with formation water. This affects the direction that the CO_2_ plume takes and how well dissolution trapping works.^38^ Natural convection can happen in the reservoir as the temperature changes, which helps CO_2_ break down into the formation water.^39,40^ As a result, accurate simulation of thermal processes is necessary for improving injection strategies, predicting long-term storage behavior, and fully assessing project risks.

#### 2.1.2 Hydraulic Process

The hydraulic process is a basic physical process in CGS that controls the complex seepage processes, pore pressure dynamics, and multiphase movement of CO_2_ inside the reservoir’s porous media.^41^ The movement of CO_2_ and formation water, which is affected by their different densities and viscosities, shows immiscible displacement properties. The geographical distribution of these qualities is determined by parameters including capillary pressure and relative permeability.^42^ This multiphase flow system determines the macroscopic migration route and sweep efficiency of the CO_2_ plume, whereas the injection process significantly alters the initial pore pressure field, creating a pressure perturbation zone centered on the injection well.^43^ The seepage behavior shows how fluids move through the pore and fracture networks of the rock. It is greatly affected by the heterogeneity of the reservoir and the density of the fractures, which together make the hydraulic processes more complicated.

A thorough comprehension of hydraulic processes is fundamentally connected to the evaluation of storage safety and efficiency. The spatial distribution of the CO_2_ plume is predominantly influenced by geological heterogeneity and fracture architecture; precise prediction of its migration trajectory is essential for guaranteeing successful sequestration within the target reservoir and preventing potential leakage routes.^43^ The increase in pore pressure resulting from injection is a critical signal for evaluating the mechanical stability of the reservoir-caprock system and the danger of induced seismicity; meticulous management is necessary to prevent overpressure from compromising the sealing integrity of the caprock.^44^ Furthermore, pore-scale interfacial phenomena (such as wettability distribution) influence the amount and spatial distribution pattern of residually trapped CO_2_ by governing capillary forces, playing a significant role in midto-long-term storage stability.^45^ Some slow physical processes (such as Ostwald ripening), although time-consuming, may also alter the distribution of the residual gas phase over long time scales and are non-negligible factors when assessing long-term storage evolution.^46^ Therefore, accurately simulating hydraulic processes is a core step in achieving everything from short-term injection control to long-term safety prediction.

#### 2.1.3 Mechanical Process

The mechanical process is central to assessing the long-term safety of CGS, involving injection-induced rock deformation, effective stress evolution, reservoir-caprock integrity, and the risk of induced seismicity.^47^ Following CO_2_ injection into deep geological formations, the resulting pore pressure increase alters the effective stress within the rock mass, which in turn governs rock deformation and potential failure.^48^ This stress change can lead to elastoplastic deformation in both the reservoir and caprock, and may even trigger the propagation of pre-existing fractures or the generation of new ones.^49^ The poroelastic reaction generated by injection can also affect strata above the reservoir and cause consequences far away from the site, such as surface uplift.^50^ As a result, it is important to measure these mechanical properties accurately in order to predict system stability, evaluate the effectiveness of caprock sealing, and stop uncontrolled deformation.

In CGS applications, mechanical risks persist throughout the whole project lifecycle, and their interconnected processes can be systematically outlined. Figure 5 shows that the rise in the reservoir pressure caused by injection is the main cause of many geomechanical dangers. If the pressure in the area surrounding the wellbore exceeds the rock’s fracture pressure, hydraulic fracturing will occur. The pressure transfer to the caprock-reservoir interface could cause the caprock to break by shear, which would put the integrity of the containment at risk. At the same time, a higher pore pressure may reactivate latent faults, which could lead to leaks of CO_2_ and produce microseismic occurrences. Also, poroelastic deformation of the reservoir will cause the underlying strata to move and the surface to rise, which could damage infrastructure on the surface. If the cement and casing in a wellbore are exposed to CO_2_ and changing stresses for a long time, then their integrity may be compromised, which could lead to leaks. The interconnected risks form a comprehensive risk chain, highlighting the necessity for extensive mechanical coupling simulation and risk assessment during the design and operational phases of a storage project.

**Figure 5:**
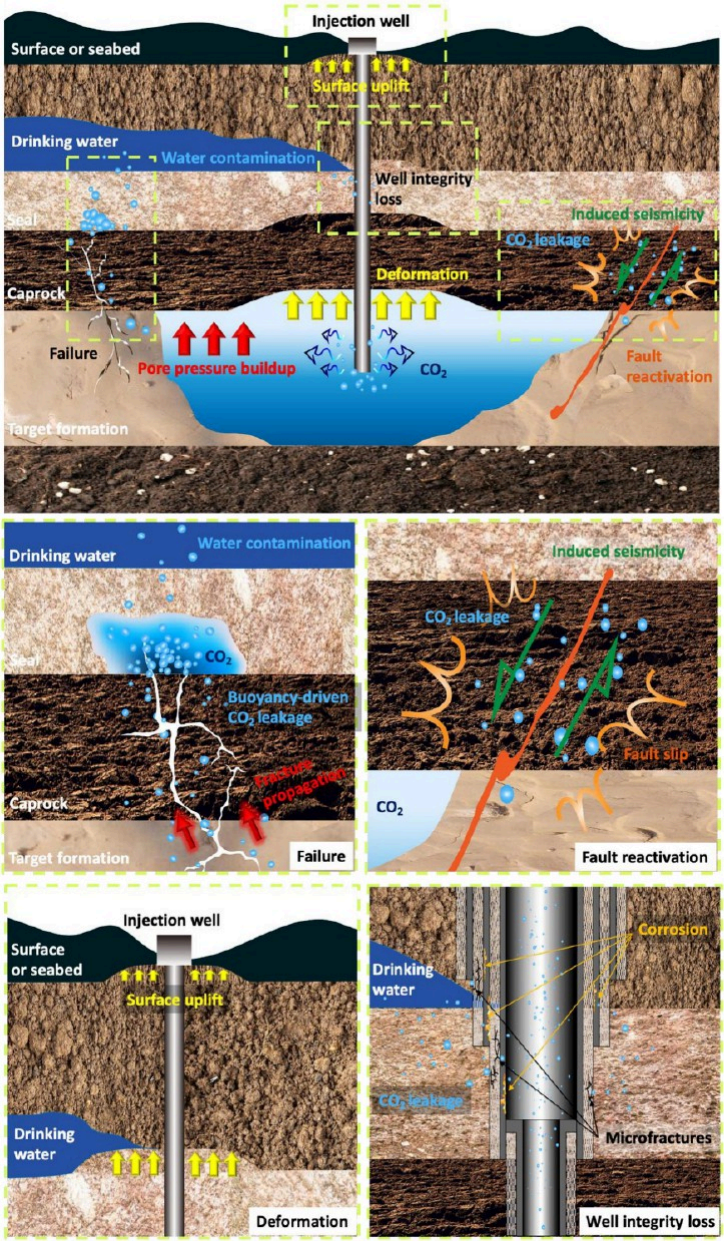
Geomechanical risks exist during the whole life cycle of a CGS project. This figure was reproduced with permission from ref 47. Copyright 2023 Elsevier.

#### 2.1.4 Chemical Process

Chemical processes are necessary for determining the long-term safety and stability of CGS, mainly the chemical interactions between the injected CO_2_, the reservoir pore water, and the mineral substrates.^51,52^ CO_2_ mixes with formation water to make carbonic acid, which lowers the pH a lot. This acidic environment makes it easier for primary silicate minerals (like feldspar and clay) and carbonate minerals (like calcite and dolomite) to dissolve.^53,54^ When minerals break down, they release cations such as Ca^2+^, Mg^2+^, and Fe^2+^. This changes the chemical makeup of pore water and may also change how porous and permeable rocks are.^55^ At the same time, the ions that are released when something dissolves may react with CO_3_^2–^ to form secondary carbonate minerals like calcite and ankerite.^56^ This precipitation is the main way to permanently store CO_2_ in minerals, but it can also block pore throats, which changes the reservoir’s properties and injectivity.^57^

In CGS, the overall effect of chemical processes, especially the fight between dissolution and precipitation, has two effects on the safety and storage efficiency. Table 1 shows that the geochemical reactions that happen when CO_2_ is injected are different, and that things like the mineral makeup of the reservoir, the temperature–pressure conditions, and the salinity of the brine have a big effect on them.^58,59^ The Supporting Information provides more specific reaction paths. Also, secondary carbonate mineralization can turn CO_2_ into a stable solid form, which is a critical step toward safe storage at the greatest level. This process has a lot of potential in reactive reservoirs like basalt.^60–62^ But chemical reactions can also be dangerous. For example, the buildup of salts or secondary minerals near the wellbore or along possible leakage pathways can make it much harder to inject fluids and store them. Mineral dissolution in the caprock or fault gouge can make it less stable, which raises the risk of leaks.^63^ Furthermore, CO_2_ that leaks into aquifers that supply drinking water can make the water more acidic, which can move contaminants such as heavy metals out of the soils and into the groundwater. This is bad for the groundwater environment.^64^ For this reason, it is very important to be able to accurately predict how chemical processes will change over time in order to figure out how the storage site will behave in the long run.

**Table 1:** Geochemical Reaction Routes and Properties of Predominant Minerals under CO_2_ Injection Conditions (Data Sources^29,65,66^ )­^a^

reaction type	reacting mineral	secondary mineral	reaction rate (mol·m^–2^·s^–1^)	chemical reaction equation
dissolution reaction	CO_2_	H_2_CO_3_, H^+^, HCO_3_^–^	fast	${\mathrm{CO}}_{2}\left(g\right)+H_{2}O\left(l\right)⇌H_{2}{\mathrm{CO}}_{3}\left(\mathrm{aq}\right)⇌H^{+}\left(\mathrm{aq}\right)+{{\mathrm{HCO}}_{3}}^{-}\left(\mathrm{aq}\right)$
	kaolinite	complete dissolution	10^–14^–10^–15^	${\mathrm{Al}}_{2}{\mathrm{Si}}_{2}O_{5}{\left(\mathrm{OH}\right)}_{4}\left(s\right)+6H^{+}\left(\mathrm{aq}\right)\to 2{\mathrm{Al}}^{3+}\left(\mathrm{aq}\right)+2{\mathrm{SiO}}_{2}\left(\mathrm{aq}\right)+5H_{2}O\left(l\right)$
	anorthite	calcite, kaolinite	1.2 × 10^–5^	${\mathrm{CaAl}}_{2}{\mathrm{Si}}_{2}O_{8}\left(s\right)+{\mathrm{CO}}_{2}\left(g\right)+2H_{2}O\left(l\right)\to {\mathrm{CaCO}}_{3}\left(s\right)+{\mathrm{Al}}_{2}{\mathrm{Si}}_{2}O_{5}{\left(\mathrm{OH}\right)}_{4}\left(s\right)$
	albite	dawsonite, quartz	–	${\mathrm{NaAlSi}}_{3}O_{8}\left(s\right)+{\mathrm{CO}}_{2}\left(g\right)+H_{2}O\left(l\right)\to \mathrm{NaAl}\left({\mathrm{CO}}_{3}\right){\left(\mathrm{OH}\right)}_{2}\left(s\right)+3{\mathrm{SiO}}_{2}\left(s\right)$
	K-feldspar	dawsonite, quartz	–	${\mathrm{KAlSi}}_{3}O_{8}\left(s\right)+{\mathrm{Na}}^{+}\left(\mathrm{aq}\right)+{\mathrm{CO}}_{2}\left(g\right)+2H_{2}O\left(l\right)\to \mathrm{NaAl}\left({\mathrm{CO}}_{3}\right){\left(\mathrm{OH}\right)}_{2}\left(s\right)+3{\mathrm{SiO}}_{2}\left(s\right)+K^{+}\left(\mathrm{aq}\right)$
	calcite	complete dissolution	1.6–3.2 × 10^–5^	${\mathrm{CaCO}}_{3}\left(s\right)+H^{+}\left(\mathrm{aq}\right)\to {\mathrm{Ca}}^{2+}\left(\mathrm{aq}\right)+{{\mathrm{HCO}}_{3}}^{-}\left(\mathrm{aq}\right),\mathrm{significant}\mathrm{mainly}\mathrm{at}\mathrm{higher}\mathrm{pH}$
precipitation reaction	calcium ion	calcite	dependent on ion activity and supersaturation	${\mathrm{Ca}}^{2+}\left(\mathrm{aq}\right)+{{\mathrm{CO}}_{3}}^{2-}\left(\mathrm{aq}\right)\to {\mathrm{CaCO}}_{3}\left(s\right)$
	ferrous ion	siderite	dependent on ion activity and supersaturation	${\mathrm{Fe}}^{2+}\left(\mathrm{aq}\right)+{{\mathrm{CO}}_{3}}^{2-}\left(\mathrm{aq}\right)\to {\mathrm{FeCO}}_{3}\left(s\right)$
	magnesium ion	magnesite	Dependent on ion activity and supersaturation	${\mathrm{Mg}}^{2+}\left(\mathrm{aq}\right)+{{\mathrm{CO}}_{3}}^{2-}\left(\mathrm{aq}\right)\to {\mathrm{MgCO}}_{3}\left(s\right)$
	calcium/sulfate ion	anhydrite	Dependent on ion activity and supersaturation	${\mathrm{Ca}}^{2+}\left(\mathrm{aq}\right)+{{\mathrm{SO}}_{4}}^{2-}\left(\mathrm{aq}\right)\to {\mathrm{CaSO}}_{4}\left(s\right)$

a Note: 1. “–” signifies that the data was either not clearly presented in the original references or is challenging to define succinctly. 2. Reaction rates are affected by multiple parameters, including temperature, pressure, pH, and mineral specific surface area; the figures in the table represent reference ranges.

### 2.2 Two-Field Coupling

#### 2.2.1 Hydraulic-Mechanical Coupling

Hydraulic-mechanical (HM) coupling is a fundamental physical mechanism in CGS, underpinned by the idea of effective stress.^67,68^ This theory asserts that variations in pore pressure directly affect the effective stress on the rock matrix, resulting in elastoplastic deformation or damage to the rock.^69^ Concurrently, rock deformation influences its internal pore structure and connectivity, resulting in changes to porosity and permeability; this stress-dependency arises from poroelastoplastic processes.^70^ Consequently, characterizing this coupling mechanism is essential for precisely forecasting the mechanical response of the reservoir and caprock.

In CGS research, HM coupling models are widely used to look at geomechanical issues and improve storage options.^71,72^ For instance, Jia et al.^73^ used both experiments and modeling to show that fluid infiltration greatly lowers fracture pressure; Bao et al.^74^ used simulations to show that viscoelastic deformation greatly increases the risk of fault instability; Ye et al.^75^ used field-scale simulations to show that caprock and surface deformation are safe in a certain block; and Chen et al.^76^ did a parameter sensitivity analysis that showed that the risk to caprock sealing integrity rises significantly when the fault dip angle exceeds 45°. These studies collectively demonstrate that meticulous attention to HM coupling is crucial for ensuring long-term safety in the design, implementation, and oversight of CO_2_ storage initiatives.^77^

#### 2.2.2 Thermal-Hydraulic Coupling

The thermal-hydraulic (TH) coupling process has a big impact on how fluids travel and how well they are trapped in CGS. Changes in temperature have a large effect on the physical properties of CO_2_, such as its density and viscosity. These things directly affect how well it flows and how pressure is distributed in porous media.^78^ At the same time, fluid flow is an effective way to move heat, and it can change the temperature distribution in a reservoir through strong convective processes.^79^ The equation of state also shows how temperature and pressure are related, which affects how injected CO_2_ behaves in different phases. Figure 6 shows that CO_2_ goes through complex phase changes from the wellhead to the reservoir. These changes cause big changes in its thermophysical properties (like density and heat capacity), which then affect the pressure in the wellbore, the temperature at the bottom of the hole, and the system’s seepage and heat transfer mechanisms.

**Figure 6:**
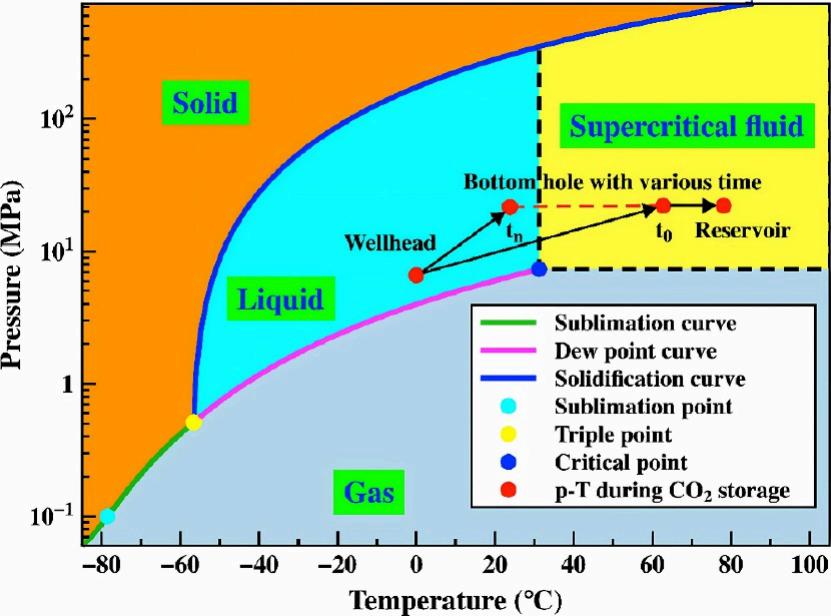
Phase changes and P-T variations during the CGS process. This figure was reproduced with permission from ref 82. Copyright 2024 Elsevier.

In CGS applications, the TH coupling effect is key to assessing plume migration, dissolution trapping, and long-term safety.^80^ For example, temperature-driven fluid density differences are the root cause of density-driven convection, which can significantly accelerate the dissolution of CO_2_ into the brine, thereby enhancing the dissolution trapping efficiency (conceptual model shown in Figure 7). However, outside the main CO_2_ plume body or in the residual gas zone, concentration gradients of dissolved CO_2_ in the aqueous phase can induce nonconvective transport dominated by molecular diffusion and deposition effects. Such slow, long-term processes may lead to the reaccumulation of CO_2_ beneath the caprock. Therefore, accurately quantifying both convective and nonconvective transport processes is crucial for predicting the spatiotemporal evolution of the CO_2_ plume, optimizing injection strategies to enhance storage benefits, and assessing long-term stability.^81^

**Figure 7:**
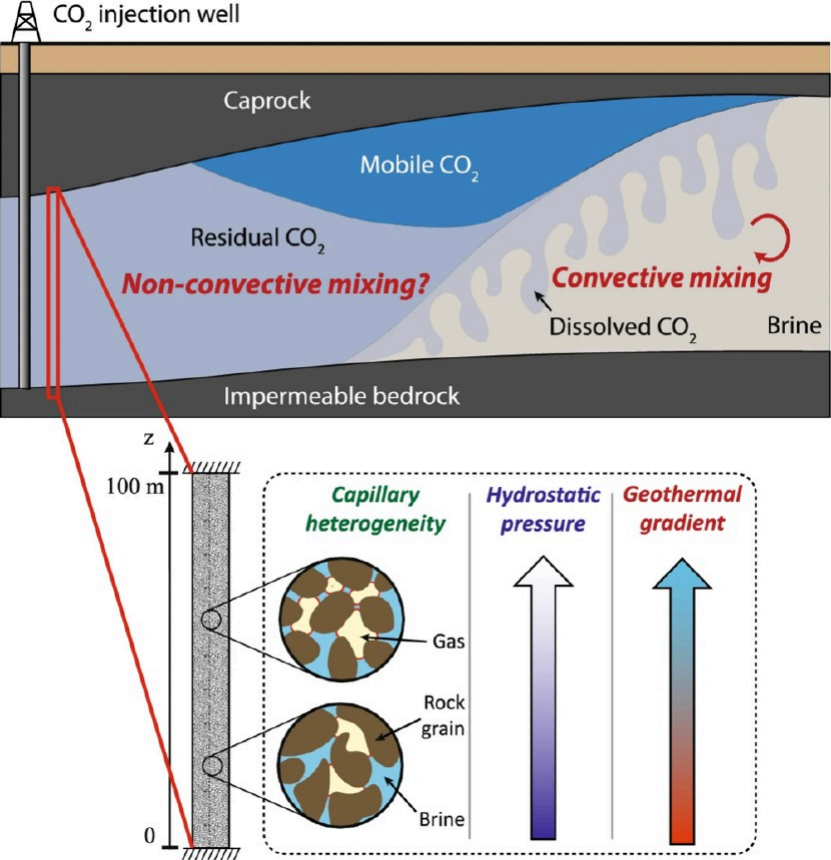
Conceptual model of CO_2_ storage in a saline aquifer, illustrating its convective mixing and nonconvective transport processes. Convective mixing occurs in the region where the migrating CO_2_ plume contacts unsaturated brine, significantly enhancing dissolution; whereas in areas lacking convection, dissolved CO_2_ migrates via nonconvective transport. This figure was reproduced with permission from ref 83. Copyright 2021 Springer Nature.

#### 2.2.3 Hydraulic-Chemical Coupling

Hydraulic-chemical (HC) coupling is a key process controlling the long-term safety and effectiveness of CGS.^84–86^ Its core lies in the interaction between fluid flow and chemical reactions: fluid flow is responsible for transporting reactants and products, controlling the spatiotemporal distribution of chemical reactions^87^; whereas reactions such as mineral dissolution and precipitation alter the structure of the porous medium (e.g., porosity, permeability), which in turn affects fluid flow paths and rates.^88^ Furthermore, complex feedback relationships exist among the CO_2_ solubility, aqueous phase composition, and reaction rates, collectively forming a dynamically evolving system, whose core reaction network is shown in Figure 8.

**Figure 8:**
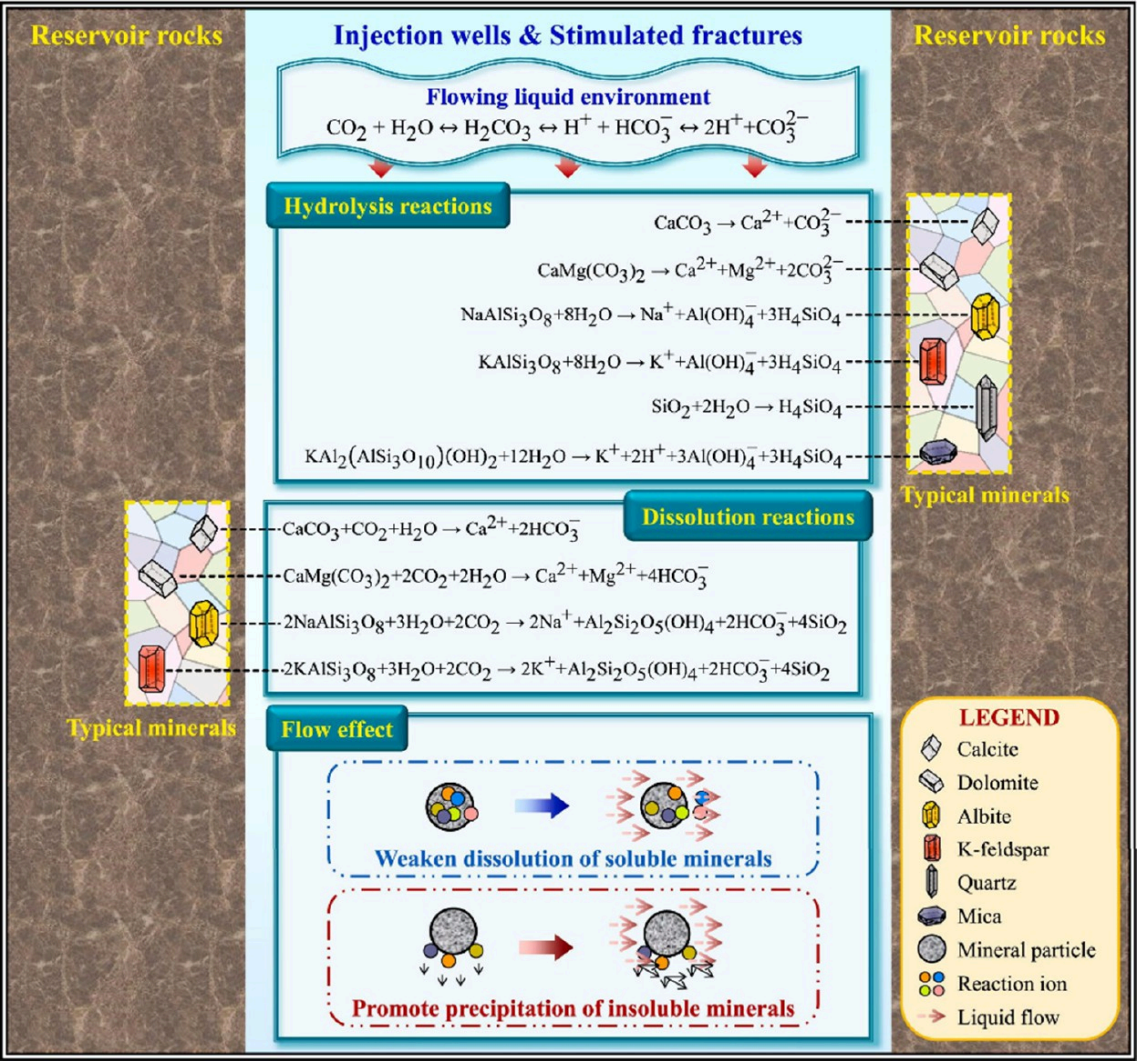
Schematic diagram of physicochemical reactions in the reservoir during CGS. This figure was reproduced with permission from ref 88. Copyright 2024 Elsevier.

In CGS, the HC coupling effect endures throughout the entire process and significantly impacts the final outcome.^89–91^ The acidic environment that forms when CO_2_ dissolves in the near-wellbore area during the injection phase may cause minerals to dissolve, which would temporarily increase the porosity and permeability. However, it might also cause secondary minerals to precipitate, which would block pores and make it harder for fluids to flow through them.^92^ Over time, mineral dissolution creates conditions that are good for mineral trapping, which is when CO_2_ reacts with formation rocks to make stable carbonates. This is one of the most reliable ways to store minerals.^93,94^ Chemical reactions can also be a problem, such as chemical erosion along faults or the caprock, possibly creating leaking channels.^95^ As shown in Figure 9, these geochemical reactions can induce a series of geomechanical effects and environmental risks. Therefore, accurately characterizing the HC coupling process is crucial for assessing the site’s mineral trapping potential, predicting pore evolution, and ensuring caprock integrity and long-term safety.^96^

**Figure 9:**
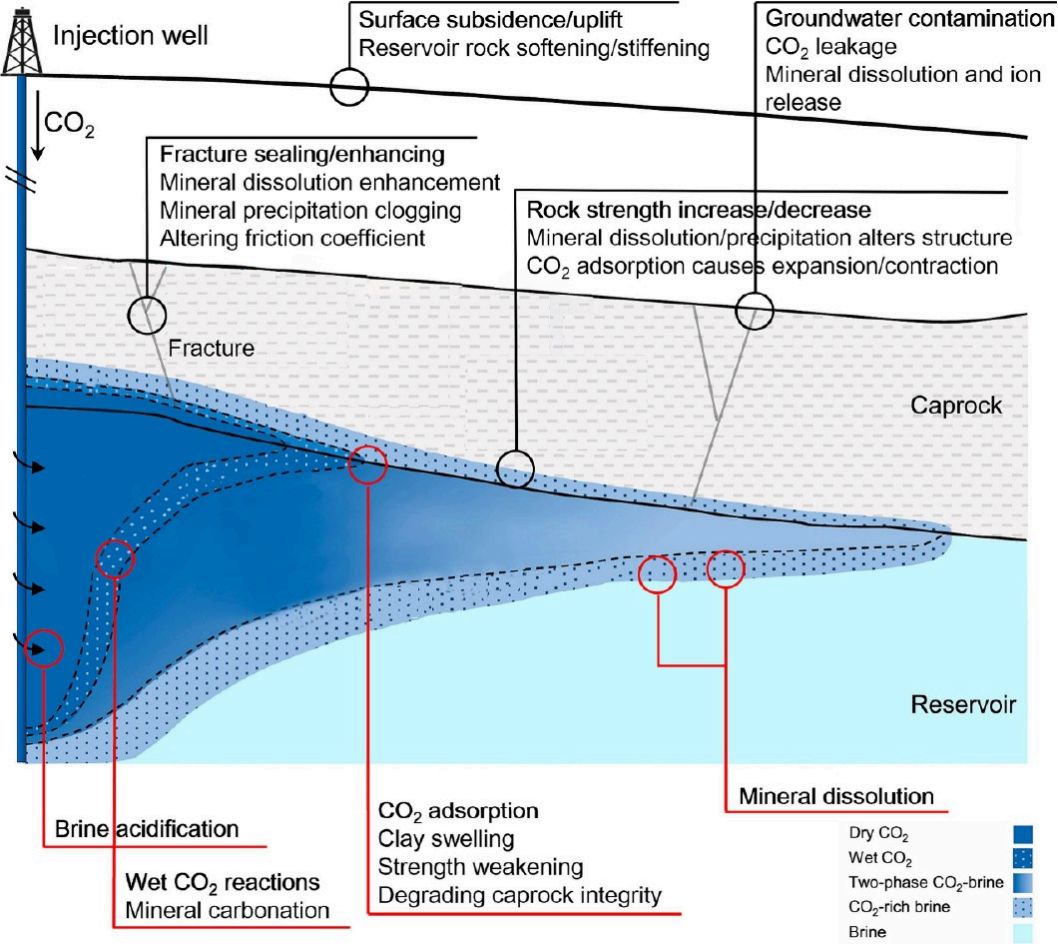
Impacts of geochemical reactions in CGS (reaction processes are colored red; associated risks in black). This figure was reproduced with permission from ref 95. Copyright 2025 Elsevier.

### 2.3 Three-Field Coupling

#### 2.3.1 Thermo-Hydro-Mechanical Coupling

Thermo-hydro-mechanical (THM) coupling is a core theoretical framework describing the complex interactions among the temperature field, seepage field, and stress field, and is crucial for accurately predicting the short-term response and long-term evolution of CGS systems.^97,98^ Its physical mechanism is as follows: the injected nonisothermal CO_2_ fluid simultaneously alters the reservoir pressure field and temperature field.^99^ Pore pressure increase directly reduces the effective stress, while the temperature difference between the cooler CO_2_ and the rock induces significant thermal stress; together, they affect the stress–strain state of the rock mass, potentially leading to elastoplastic deformation or shear failure of the rock.^100^ Such mechanical deformation, by altering the pore structure and fracture network, feedbacks to affect the macroscopic properties of the reservoir (e.g., porosity, permeability), forming a dynamic closed loop: fluid flow and heat transport drive mechanical deformation, and the deformed medium in turn constrains the efficiency of fluid flow and heat conduction, exhibiting complex spatiotemporal evolution characteristics in the near-wellbore region.^101^ These tightly interwoven processes constitute a complete THM coupling system, whose interaction mechanism is shown in the conceptual model in Figure 10, systematically illustrating the complete feedback loop from gas migration, competitive adsorption, rock deformation to heat transfer and property evolution.^102^ A deep understanding of this coupled system is the foundation for assessing reservoir injectivity, predicting CO_2_ plume migration, managing geomechanical risks, and ensuring long-term storage safety.

**Figure 10:**
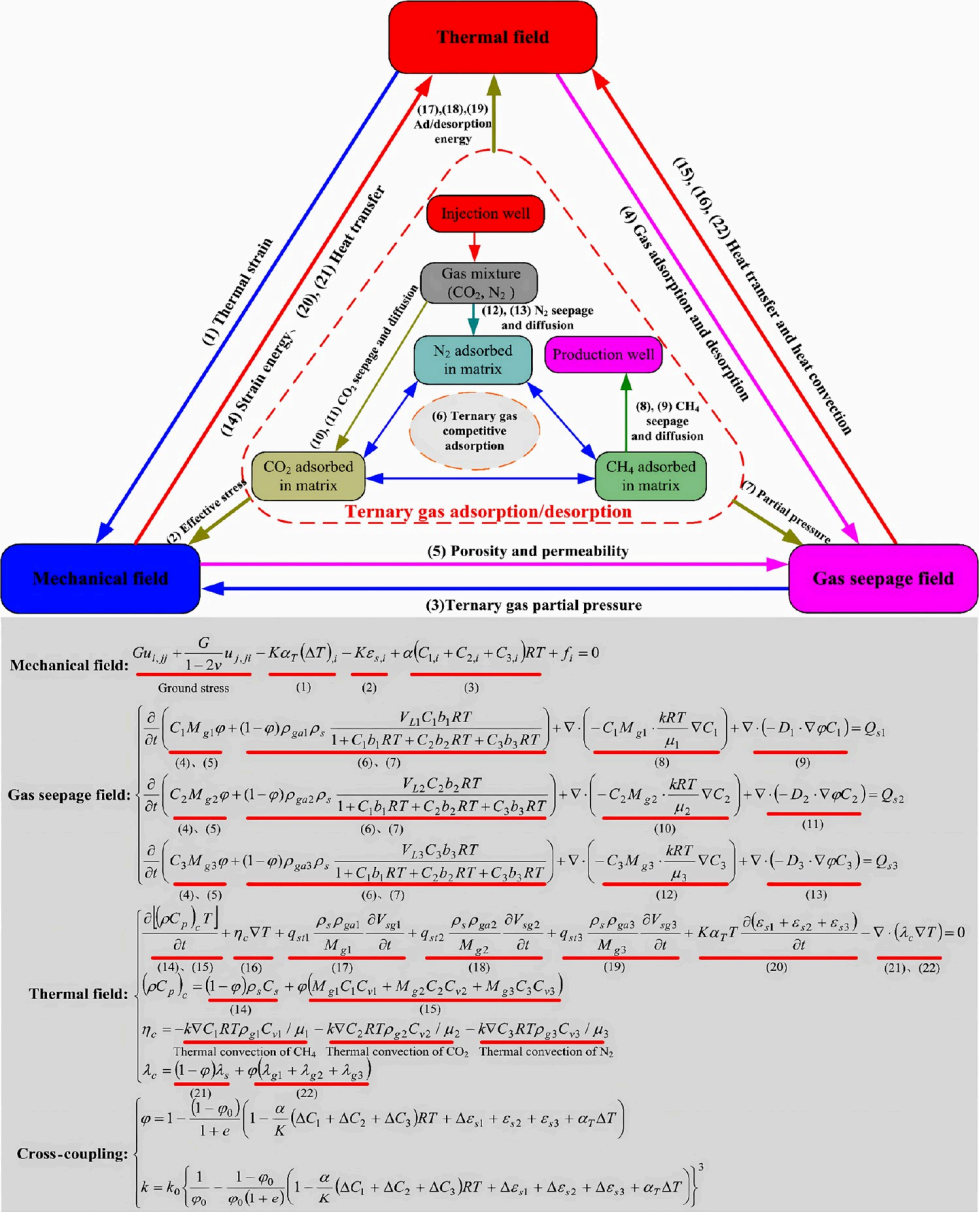
THM coupling model shows how mixed gas can be injected into coal seams by coupling. This figure was reproduced with permission from ref 102. Copyright 2019 Wiley.

In CGS applications, THM coupling models are widely used for optimizing schemes and assessing risks. For example, Liu et al.^103^ simulated and confirmed that increasing injection pressure can simultaneously enhance CH_4_ recovery (4.26–12.80%) and CO_2_ storage capacity (per well 0.73–2.54 × 10^5^ m^3^). In shale gas reservoir applications, Cheng et al.,^104^ using a self-developed THM coupling model, found that strong in situ stress anisotropy significantly increases the amount of free CO_2_ trapped in shale, and revealed the dynamic characteristics of matrix and fracture porosity. Regarding safety assessment, Ye et al.^105^ integrated a mechanical module into the TOUGH2 simulator, predicting that the pressure influence zone (10 km) in the Ordos CCS project is much larger than the CO_2_ plume (620 m) and the low-temperature zone (100 m), and accurately assessed surface uplift (maximum 0.14 m) and the spatiotemporal evolution of effective stress. Wang^106^ based on THM coupling theory, established an analytical model including conduction-convection heat transfer, analyzed the temperature and pressure disturbance patterns in formations with different permeabilities under CO_2_/CH_4_ injection, and revealed the impact of supercritical phase transition and thermal flow characteristics on storage capacity. Li et al.,^107^ using a phase-field THM model, confirmed that CO_2_ fracturing can create a more complex fracture network due to the thermal stress effect, providing new insights for reservoir stimulation.

Previous research has achieved systematic progress in THM coupling. Theoretically, methods ranging from analytical solutions to complex numerical models have been developed, deepening the understanding of mechanisms such as thermo-poroelastic response.^108,109^ Studies have investigated various reservoirs, including coal seams, shale formations, and saline aquifers, identifying key controlling factors such as competitive adsorption, in situ stress anisotropy, and thermal stress, while also quantifying the synergistic benefits of CO_2_ storage and energy extraction (e.g., ECBM).^110^ THM coupling analysis has become a key tool for predicting pressure propagation, surface deformation, microseismic activity, and fracture propagation in safety evaluations. This gives scientists a solid basis for choosing a site, optimizing injection, and monitoring over time.^111^ Future research will concentrate on developing efficient, fully coupled simulators and improving the integration of THM factors with chemical effects to provide a more comprehensive predictive framework.

#### 2.3.2 Hydro-Chemical-Mechanical Coupling

Hydro-chemical-mechanical (HMC) coupling describes the complex interactions between fluid dynamics, chemical processes, and rock stress deformation. It is a key theoretical basis for assessing the long-term safety and effectiveness of CGS. The basic process is the acidic fluid that forms after CO_2_ is injected. This fluid controls how reactants move around and where they go, which affects the site and rate of chemical reactions between water and rock, such as mineral dissolution and precipitation.^112^ Chemical reactions change the structure of the pore media right away. For example, mineral dissolution can make pores wider, while secondary mineral precipitation might block pore throats. This change in porosity and permeability has a big effect on the reservoir’s flow capacity and pressure distribution.^113^ Chemical operations change the mechanical properties of the rock at the same time. Changes in pore structure and fluid pressure fluctuations affect the stress state of the rock mass through the effective stress principle, which could cause deformation or fault reactivation.^114^ On the other hand, changes in the stress field (such as rock deformation or fault sliding) change the crack aperture and connectivity, creating new transport paths or sealed zones for fluids and solutes. This changes the pathways and extent of chemical reactions.^115^ The robust bidirectional feedback within HMC forms a highly nonlinear dynamic system, necessitating the creation of intricate multifield coupling computational frameworks to concurrently resolve the governing equations for fluid dynamics, solute transport, chemical reactions, and solid deformation.^116^ A comprehensive understanding of HMC coupling is essential for forecasting the long-term development of CO_2_ storage systems, especially in evaluating the caprock integrity, fault stability, and mineral trapping efficacy.

In CGS application research, HMC coupling models are widely used to quantify storage mechanisms and assess geomechanical risks. In unconventional reservoirs, Cai et al.^112^ established an HMC model coupling fluid phase behavior, multicomponent flow, reservoir deformation, and reaction-controlled pore evolution, finding that nanoconfinement effects can alter component chemical potential, reduce CO_2_ solubility and minimum miscibility pressure, while increasing heavy component production and CO_2_ retention rate, thus optimizing tight reservoir storage and production design. Cai et al.^115^ further constructed a fully coupled model incorporating static and dynamic microscale effects, using an embedded discrete fracture model to represent the matrix-fracture system, and systematically analyzed the comprehensive impacts of micronano pore proportion, minimum miscibility pressure, and stress sensitivity on CO_2_ enhanced oil recovery and storage in tight reservoirs. Regarding fault stability, Tounsi et al.^117^ simulated CO_2_ storage in the Dogger formation of the Paris Basin using an HMC model and found that calcite-rich faults have a low risk of reactivation over the long-term (approximately 100 years), providing an effective tool for fault stability assessment in such sites. Yan et al.^114^ conducted HMC-coupled fault reactivation research using a unified pipe-interface element method; numerical simulations indicated that caprock faults are more susceptible to reactivation than reservoir faults, and in long-term CO_2_ leakage scenarios, the influence of chemical reactions on fault slip is more significant than that of fluid pressure. It is noteworthy that the relative importance of each coupling process varies under different site conditions. For instance, simulations by Varre et al.^118^ showed that in specific scenarios, the impact of mineral dissolution and precipitation on reservoir porosity and macroscopic mechanical response (e.g., surface displacement) might not be significant. Collectively, these studies demonstrate that HMC coupling analysis is the cornerstone for assessing the long-term effectiveness and safety of CO_2_ storage, and the development and application of its models provide a key scientific basis for site screening, risk assessment, and optimization management.

#### 2.3.3 Thermo-Hydro-Chemical Coupling

In CGS, Thermo-hydro-chemical (THC) coupling is a key process that reveals the complex interactions among the temperature field, fluid flow, and chemical reactions.^119^ Its core mechanism lies in CO_2_ injection, which alters the reservoir temperature field, and temperature changes not only regulate fluid viscosity, density, and flow behavior but also significantly affect the kinetic rates and equilibrium states of chemical reactions such as mineral dissolution and precipitation.^120^ However, current multifield coupling research often focuses on dual-field couplings such as HM or HC, while studies on complete THC coupling, particularly the two-way feedback between the temperature and chemical fields, are still insufficiently deep. Many models treat temperature as a passive outcome or a fixed parameter, failing to adequately couple the thermal effects of chemical reactions and thereby limiting the accurate prediction of reservoir geochemical evolution during long-term storage. For example, Li et al.,^121^ while studying the coinjection of CO_2_ and SO_2_, established a THC model that revealed the dominant role of the chemical field: the injected gases react with formation water to create an acidic environment, leading to mineral dissolution and significantly altering reservoir porosity. This work successfully documented the strong HC relationship; however, the examination of the dynamic interaction between the temperature field and intense chemical processes was inadequate. This is a good example of a common problem in modern THC research: while chemical processes are well-described, the way they change with temperature changes has not been clearly defined, which could make it harder to accurately assess the long-term stability of the storage system.

### 2.4 Four-Field Coupling

THMC coupling is the core theoretical framework describing the complex interactions among the four fields: temperature, fluid flow, mechanical response, and chemical reactions in CGS. Its fundamental principle is that changes in one physical field can trigger chain reactions in the others.^16^ For example, CO_2_ injection causes changes in pore pressure and temperature, which alter the rock mass stress state via the effective stress principle and thermal stress; stress changes induce microfracture propagation, increasing reactive surface area and accelerating mineral dissolution or precipitation; ultimately leading to fundamental alterations in reservoir porosity, permeability, and mechanical strength. This complex feedback cycle has a decisive impact on the long-term safety of the storage system.^122^ Table 2 systematically reviews representative studies of THMC multifield coupling in CGS in recent years.

**Table 2:** Representative Studies of THMC Multi-Field Coupling in CGS

application scenario	coupling method and model	key findings and mechanisms	engineering implications and risk assessment	references
supercritical CO_2_ injection into saline aquifers	a fully coupled THMC model was developed for numerical simulation	low-temperature heat conduction significantly affects the mean stress and chemical reaction rate; geochemical reactions have a minor impact on pore pressure and temperature	revealed the differential influence weights of the multiphysics fields, indicating that THM coupling is the key interaction	^125^
supercritical CO_2_ as geothermal fluid	developed a THMC-coupled model to study injection and reservoir interactions	mineral dissolution–precipitation kinetics significantly affect rock permeability; Injection over 6.34 years can sequester substantial CO_2_ while enhancing reservoir permeability and heat extraction efficiency	confirmed the feasibility of synergizing CO_2_ storage with enhanced geothermal systems, achieving a “storage-heat extraction” win-win	^124^
injection test at the Cranfield Site, USA	high-resolution 3D heterogeneous model, using CMG-GEM for sequential coupling of THMC processes	neglecting thermal effects leads to underestimation of bottom-hole pressure and residual trapping, while overestimating dissolution trapping; Ignoring capillary pressure heterogeneity underestimates dissolution trapping	emphasizes the need for accurate consideration of heat transport and capillary effect heterogeneity in field-scale predictions	^16^
horizontal well storage test in Mikołów, Poland	utilized a THMC dual-porosity model for preoperational simulation	adjusting horizontal well length and injection conditions enables sustained injection without significant permeability reduction, with controllable plume extent	provides a design basis and operational window for the safe and controllable injection of CO_2_ via horizontal wells	^18^
caprock integrity for supercritical CO_2_ storage	THMC coupled numerical simulation	continuous gas injection induces fault reactivation, leading to leakage; competition between mineral dissolution and precipitation causes a slight increase in fault zone permeability; Feldspar dissolution leads to a significant increase in ion concentration	reveals that fault zones possess concurrent “self-sealing” potential and failure risk, governed by the evolution of porosity and permeability	^123^
carbon mineralization in fractures of mafic/ultramafic rocks	combined experimentation and modeling to develop a new THMC coupling model	fracture aperture, flow characteristics, and surface features have a decisive influence on the quantity and morphology of secondary precipitates	provides a key mechanistic model and technical pathway for achieving permanent storage through accelerated mineralization	^122^
gas production from hydrate reservoirs and CO_2_ reinjection	established a THMC multifield coupling model	CO_2_ reinjection for remediation can achieve formation subsidence recovery, enhance mechanical performance, and enable carbon storage via hydrate formation	proposes a novel integrated “production-storage-remediation” concept, balancing energy extraction, storage, and geomechanical safety	^19^
geomechanical risk assessment for CO_2_ storage	based on a THMC coupling model, employed Monte Carlo and Sobol sensitivity analysis	generated 616 cases; 90.1% of cases exhibited surface uplift <8 mm; Reservoir permeability of 1 mD readily induces >100 mm uplift and rock failure	provides a systematic risk assessment methodology, identifying reservoir permeability as the most critical parameter for geomechanical stability	^27^

THMC coupling models significantly enhance the predictive and control capabilities for engineering problems. In the caprock integrity assessment, Chen et al.^123^ simulated and showed that injection may reactivate faults, and the “self-sealing” behavior of faults depends on the competition between mineral dissolution and precipitation, which is a typical THMC process. Regarding the integration of storage with energy extraction, Gan et al.^124^ confirmed that using supercritical CO_2_ as a working fluid can significantly enhance reservoir permeability and heat extraction efficiency through mineral reactions, achieving a win-win situation for storage and geothermal development. Additionally, Masum et al.^18^ developed a THMC model to establish a foundation for optimizing horizontal well injection parameters, hence assuring a safe and manageable injection procedure.

In conclusion, the THMC multifield coupling study is essential for precisely forecasting the long-term performance and safety of CGS. It can explain how individual processes work and how complex phenomena happen when several fields interact, such as fault reactivation, self-sealing effects, and changes in reservoir parameters. Although THMC coupling simulation still faces challenges such as high computational cost, it provides an indispensable theoretical tool and decision support for risk assessment, optimal design, and long-term monitoring of storage projects.

### 2.5 Perspectives and Prospects

Despite notable advances in THMC coupling research, the coupling mechanisms vary significantly across different reservoir conditions, and a systematic understanding of the underlying principles remains lacking. In saline aquifers, the initial transport and dissolution of CO_2_ are frequently regulated by thermal-hydraulic coupling, attributed to heightened fluid saturation and sluggish mineral reactions, especially under pronounced temperature-gradient-induced density convection. On the other hand, HC coupling controls the long-term development. This occurs when minerals dissolve and precipitate over time, changing the pore structure and altering the integrity of the containment and long-term stability. In depleted gas reservoirs with a low initial pressure and a very stable pore structure, HM coupling usually plays a big role. The pressure changes that happen when you inject something and the differences in effective stress that follow have a direct effect on the integrity of the caprock and the chance of fault reactivation. The interaction between CM and HC coupling is especially important in fractured reservoirs, such as shale, tight sandstone, or basalt. The fracture network makes it easier for fluids and solutes to move around and increases the reactive surface area. So, mineral dissolution can change the size of a fracture (either by opening or closing it), and the mechanical feedback that comes from this can change the flow patterns and reaction routes even more.

A primary challenge in current THMC coupling research is quantifying the intensity of the highly nonlinear feedback and discerning the dominating processes among the four physical fields. Current models frequently rely on constitutive relationships that are too simplistic, making it hard to find tipping points and rapid changes in behavior in real-world situations. The bidirectional interaction between chemical and mechanical fields, exemplified by stress corrosion and reaction-induced deformation, represents a theoretical vulnerability: while mineral precipitation can enhance strength, it may simultaneously induce microfracturing due to crystallization pressure; these opposing mechanisms are inadequately integrated into existing models. Simultaneously, while the Arrhenius effect of the thermal field on reaction rates is often considered, the local temperature changes induced by the reaction heat and their feedback to the flow and stress fields are frequently neglected, constraining long-term prediction accuracy. Future mechanistic research needs to develop more refined constitutive theories, particularly mathematical models capable of describing the intrinsic linkages among medium deformation, seepage, and reaction under fully coupled THMC pathways, to reveal the cross-scale coupling mechanisms from the pore to the field scale. Figure 11 systematically summarizes the challenges and future directions for the THMC coupling mechanisms.

**Figure 11:**
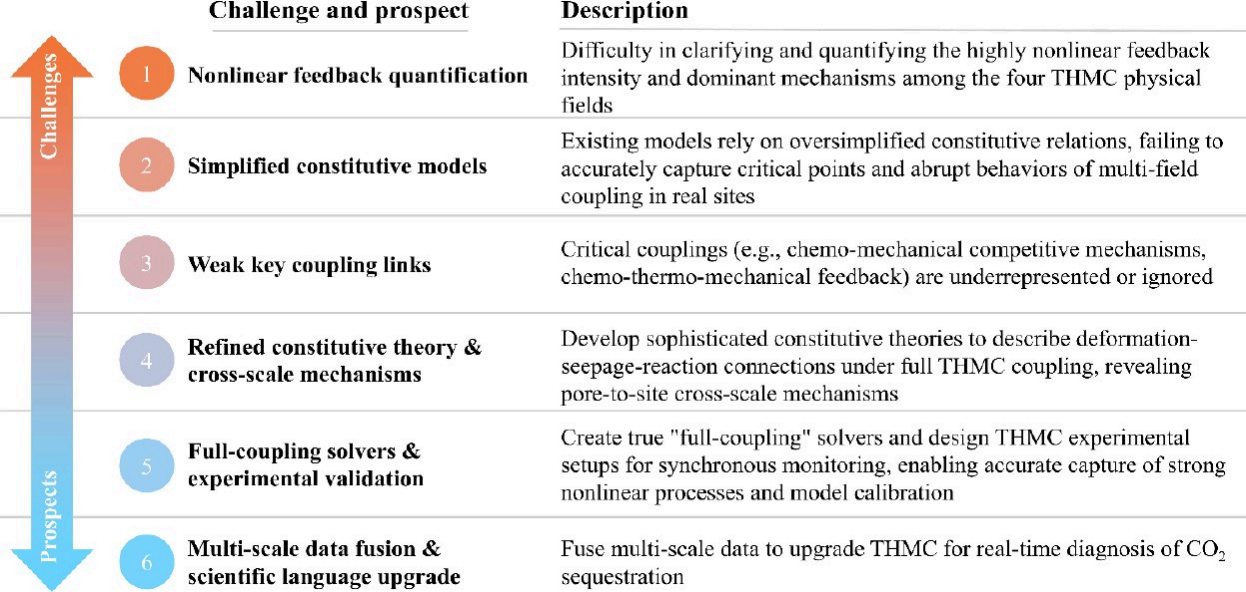
Current issues and future advancements in THMC coupling mechanisms.

To break through the aforementioned bottlenecks, future research should emphasize methodological innovation and the integration of multisource data. In terms of computational methods, there is a need to develop genuinely “fully coupled” solvers to avoid the nonconservation of energy and mass caused by sequential coupling, enabling the precise capture of strongly nonlinear transient processes.^126^ For experimental validation, there is an urgent need to design THMC coupling apparatuses capable of simultaneously monitoring temperature, pore pressure, deformation, and fluid chemistry, to provide benchmark data for model calibration, particularly for the coupled behavior at key interfaces such as faults and caprocks.^123^ In the end, it is important to use a multiscale simulation framework to connect the changes in pore structure seen through micro-CT with core experimental parameters and field-scale geophysical responses (like seismic wave velocity and electrical resistivity). This will create a geophysical response map that shows how the THMC coupling works. This will change THMC coupling from a complicated numerical tool into a scientific language that can be used with field monitoring data for real-time diagnostics. This will finally make the mechanisms clear and allow for accurate predictions of how the CGS will evolve over time.

## 3 THMC Coupling Methods

### 3.1 Coupling Strategies

In the multiphysics simulation of CGS, the main ways to couple numbers are one-way coupling, sequential coupling, and full coupling.^127^ One-way coupling is a simple method in which data flows only in one direction. The results of earlier fields (like flow field) are used to calculate later fields (like mechanical or chemical fields) without taking feedback into account, which means that interactions between fields are not taken into account.^128^ Sequential coupling allows for bidirectional data transfer between fields by sequentially solving the governing equations of each physical field within a single time step and iterating until convergence, thereby approximately achieving coupled feedback.^129^ The fully coupled strategy consolidates the governing equations of all physical fields into a single system and solves them simultaneously at each time step, theoretically enabling the most complete and stable capture of instantaneous nonlinear interactions between fields.^130^ These three strategies have distinct characteristics in terms of computational efficiency, accuracy, and implementation complexity, with their core differences summarized in Table 3.

**Table 3:** Comparison of One-Way, Sequential, and Fully Coupled Strategies in CGS Simulation

characteristic dimension	one-way coupling	sequential coupling	full coupling
core principle	unidirectional data transfer, no feedback	fields solved separately, bidirectional feedback achieved through iteration	governing equations of all physical fields are assembled and solved simultaneously
data flow	unidirectional, linear	bidirectional, iterative loop	fully interwoven, highly nonlinear
computational efficiency	high	medium (depends on the number of iterations and the convergence criteria)	low
numerical accuracy	low (key feedbacks ignored)	medium to high (depends on iterative convergence)	high (theoretically most accurate)
implementation difficulty	low (can utilize existing standalone simulators)	medium (requires managing data exchange and iterative workflow)	high (requires developing specialized solvers)
numerical stability	generally stable (no feedback loops)	may face risk of iterative nonconvergence	extremely high demands on nonlinear solvers, potentially unstable
resource demand	low	medium	very high (memory and computation)
capability to capture nonlinear feedback	weak	medium to strong	strong
typical application scenarios	preliminary screening, long-term risk scanning, and systems where coupling effects are not dominant	most detailed engineering design and risk assessment, where coupling feedback is important but not extreme	study of strongly nonlinear transient processes (e.g., near-wellbore, fault reactivation, fracture dynamics), mechanistic research, benchmark solution generation

Due to its high computational efficiency, one-way coupling is often applied in scenarios where coupled feedback is insensitive or for large-scale preliminary risk assessment. For example, when assessing injection-induced regional geomechanical stability, both Vidal-Gilbert et al.^131^ and Roehmann et al.^132^ employed the one-way coupling method, transferring the pressure field obtained from flow simulation as a fixed input to the geomechanical model, successfully evaluating the risks of surface uplift and fault slip; the results indicated controllable risks in these specific cases. Similarly, research by De Lucia et al.^128^ confirmed that one-way decoupling of fluid dynamics from chemical reactions in CO_2_–water–rock reaction systems can significantly improve computational efficiency while maintaining acceptable accuracy, making it suitable for long-term chemical evolution prediction.

Sequential coupling offers a good balance between computational cost and simulation accuracy, leading to its widespread application in CGS research.^133,134^ This method can capture key feedback mechanisms such as stress changes affecting permeability or chemical reactions altering pore structure. Wang et al.,^135^ by sequentially coupling a permeability-stress model with the TOUGH2Biot simulator, quantitatively revealed that mechanical effects can increase the lateral migration distance of CO_2_ by 13.1% and enhance the storage capacity by 11.6%. Ju et al.,^136^ by sequentially integrating a hydraulic fracturing module with a THM model, investigated the fracture propagation behavior induced by CO_2_ injection and analyzed the impact of thermal contraction on fracture vertical extension, providing important insights for injection safety.

The fully coupled strategy is suitable for highly nonlinear, transient processes or simulation scenarios requiring extremely high accuracy.^137^ This method can rigorously handle the tight interactions between various physical fields, offering significant advantages in simulating near-wellbore behavior, fault reactivation, and fast chemical reaction processes. Ahusborde et al.^138^ specifically developed a fully coupled implicit finite volume method for solving the coupled problem of two-phase flow and geochemical reactions, verifying the accuracy and robustness of this method in CGS simulation. Research by Beck et al.^127^ pointed out that under transient multiphase flow conditions, iterative sequential coupling schemes require a sufficient number of iterations to achieve numerical accuracy comparable to fully coupled schemes, further highlighting the necessity of full coupling in complex scenarios.

### 3.2 Numerical Methods

In the multiphysics coupled simulation of CGS, the choice of numerical method directly affects the accuracy and reliability of the computational results. The finite element method (FEM), based on the variational principle, tackles the weak form of governing equations through element discretization and shape function approximation, making it suitable for complex geometric boundaries and solid mechanics problems.^139,140^ The finite volume method (FVM) uses control volume integration to ensure that the mass, momentum, and energy are conserved locally. This makes it great for simulating reactive transport and multiphase flow.^141,142^ The finite difference method (FDM) quickly turns differential terms into differences, which makes it easy to use and easy to compute on uniform grids. Additionally, the discrete fracture network (DFN) approach clearly shows fracture systems, the discrete element method (DEM) shows how material behaves mechanically at the particle level, and computational fluid dynamics (CFD) focuses on simulating complicated fluid dynamic processes.^143^ Table 4 shows the essential features and areas of application for each of these techniques. Each one has its own set of traits that makes it useful for THMC-linked simulations across a range of sizes and physical processes.

**Table 4:** Comparison of Major Numerical Methods for THMC Simulation in CGS

method dimension	FEM	FVM	FDM	DFN	DEM
core principle	based on the variational principle, solves the weak form of governing equations via element discretization and shape function approximation	integrates conservation equations over control volumes, strictly ensuring local conservation of physical quantities	directly approximates differential equations using difference formulas on regular grids	explicitly represents fracture systems as discrete lower-dimensional geometric elements (e.g., polygons)	models the medium as a collection of discrete particles, solving their interactions and motion based on contact mechanics and Newton’s laws
coupling capability	strong, particularly excels in mechanical coupling	strong, specializes in flow-transport coupling	medium, suitable for simple coupling	specializes in fluid–solid coupling	specializes in particle-scale coupling
computational efficiency	medium to Low, computational cost increases significantly with problem size and nonlinearity	high, performs exceptionally well in convection-dominated flow and transport problems, easily parallelized	high, fastest computational speed on regular grids and for simple problems	medium, computational cost is highly influenced by the complexity and density of the fracture network	very low, computational overhead increases exponentially with the number of particles, typically limited to micro scales
primary applicable scale	laboratory to field scale (e.g., reservoir deformation, regional stress analysis)	pore to field scale (e.g., large-scale fluid migration simulation)	laboratory to field scale (especially when the geometry is regular)	fracture to the reservoir scale	particle to laboratory scale
advantages	strong capability in handling complex geometries and boundary conditions	possesses strict local and global conservation properties	intuitive principle, simple program implementation	can explicitly represent discontinuous media, accurately describing fracture control on seepage and stress	can directly simulate discontinuous processes like fracture and particle migration
	mature theoretical framework for solid mechanics and multifield coupling	robust and stable for solving flow, heat transfer, and transport problems	highest efficiency in regular computational domains	effectively characterizes strong anisotropy	reveals the microscopic mechanisms behind macroscopic mechanical behavior
	ease of implementing high-order accuracy schemes	facilitates large-scale parallel computation			
limitations	high computational resource consumption	less convenient than FEM for complex solid mechanics simulations	accuracy decreases significantly when handling complex geometries, often requiring complex grids	fracture geometry and mechanical parameters are difficult to obtain accurately	extremely high computational cost
	conservation properties for strongly nonlinear, convection-dominated problems are inferior to FVM	achieving high-order accuracy is more complex than in FEM	conservation is not easily strictly guaranteed	computational cost increases dramatically with the number and connectivity of fractures	difficult to apply directly to engineering scales, requires coupling with continuum methods
typical applications	caprock integrity, fault reactivation, surface uplift, fully coupled THMC process analysis	CO_2_ plume migration within reservoirs, temperature field evolution, dissolution trapping, chemical reaction transport	rapid analysis of flow and heat transfer in regular conceptual models, and hydrate formation simulation	fractured reservoir assessment, identification of preferential migration paths, leakage risk along faults/fractures	evolution of rock micromechanical properties, degradation due to CO_2_-water-rock interactions, and sand production mechanisms
reference cases	^144,151^	^138,152^	^39^	^145^	^146,153^

In real-world situations, people use a lot of different numerical methods to solve important problems in CGS since each one has its own benefits. The FEM is widely used to study geomechanical reactions such as caprock integrity, fault reactivation, and surface deformation since it is better at analyzing solid mechanics. For example, Arjomand et al.^144^ developed a three-dimensional finite element model to show how CO_2_ injection at the In Salah site changed the shape of the surface. The FVM is necessary for modeling the movement of several fluids and chemical reactions. Ahusborde et al.^138^ successfully simulated the interplay between two-phase flow and geochemical processes. The DFN method is very useful for looking at fractured reservoirs. Niyogi et al.^145^ used a DFN model to look at the permeability and storage capacity of dykes in the Deccan Volcanic Province. The DEM can clarify micromechanical mechanisms. Zhang et al.^146^ examined the effects of CO_2_ injection on the mechanical characteristics of limestone via DEM simulations. CFD is essential for safety risk assessment; Wang et al.^147^ utilized CFD to model the leakage and dispersion of CO_2_ from high-pressure pipelines. The previously stated applications illustrate the benefits of various strategies in addressing these particular issues.

The THMC-linked simulation for CGS necessitates the selection of suitable numerical methods contingent upon the study aims and scale. Optimally, a combination of methodologies should be employed synergistically: FEM addresses mechanics-dominated issues, FVM manages flow-dominated challenges, DFN effectively delineates fracture networks, DEM uncovers microscopic mechanisms, and CFD is appropriate for particular fluid dynamic phenomena.^148–150^ Future research must concentrate on creating multimethod coupled cross-scale simulation frameworks that amalgamate microscopic mechanisms with macroscopic behavior, improve computational efficiency, and consequently predict the long-term evolution of CO_2_ storage systems with greater accuracy, thereby offering theoretical support and a foundation for decision-making in engineering practice.

### 3.3 Coupling Software

The long-term safety and efficacy assessment of CGS highly depends on the accurate numerical simulation of coupled THMC processes.^154–156^ Currently, various mature coupled simulation platforms provide core tools for revealing THMC interactions by integrating key processes such as multiphase flow, heat transport, mechanical deformation, and geochemical reactions.^157–159^ These platforms are mainly divided into two categories: one comprises comprehensive simulators with modular architectures like the TOUGH series, CMG, and ECLIPSE, which typically achieve coupling analysis for specific scenarios through embedded or extended interfaces^160–162^ ; the other category consists of multiphysics simulation environments represented by COMSOL and OpenGeoSys, which support user-defined coupling processes.^39,163^ To systematically review this technology landscape, Table 5 compares mainstream THMC simulation software from perspectives such as core functionality, primary coupling fields, and typical applications, aiming to assist researchers in selecting appropriate tools based on specific needs (e.g., long-term mineral trapping, caprock integrity, or leakage risk assessment).

**Table 5:** Overview of THMC Simulation Software and Coupling Schemes for CGS

primary platform/software series	specific module/coupling scheme	core functionality	coupling application (primary fields)	typical application in CGS	references
TOUGH series	TOUGHREACT (and its variants)	geochemical reaction module, simulates water-rock interactions	HC, THC	long-term mineral trapping, reservoir property evolution, impact of impure gas injection, and biogeochemical processes	^186–188^
	TOUGH2/ECO2N	THM module for CO_2_-brine systems	TH	CO_2_ plume migration, dissolution trapping, pressure buildup, and large-scale industrial simulation	^189,190^
	TOUGH2/EOS7C	simulates CO_2_ migration and CO_2_–CH_4_ mixing in depleted gas reservoirs	TH	depleted gas reservoir storage, Joule-Thomson effect, CBM production and storage	^191,192^
	T2Well/ECO2N (ECO2M)	wellbore-reservoir coupled simulation	TH (wellbore-reservoir)	wellbore temperature/pressure dynamics, injectivity analysis, intermittent eruption mechanisms	^26,193,194^
	TOUGH2Biot	TOUGH2 extension incorporating simplified mechanical calculation	THM	preliminary geomechanical response, surface uplift estimation, shear failure assessment	^195^
	iTOUGH2	uncertainty quantification and parameter inversion	(consistent with parent module)	model calibration, sensitivity analysis, leakage risk prediction	^196^
	TOUGH2/TMVOC	simulates comigration of multicomponent volatile organic compounds and CO_2_	TH (multicomponent)	shallow aquifer leakage risk assessment, multicomponent gas migration	^197,198^
	TOUGH-FLAC3D	coupling of TOUGH2/3 with mechanical software FLAC3D	THMC	caprock integrity, fault reactivation, surface deformation, induced seismicity analysis	^199–201^
	TOUGH-PyLith	coupling of TOUGH2 with open-source FEM software PyLith	THM	fault mechanical behavior, regional stress field changes	^202^
	TOUGH-FrontISTR	coupling of TOUGH2 with open-source FEM code FrontISTR	THM	environmental impact assessment, site-scale coupled simulation	^203^
	TOUGH2-Code_Aster	coupling of TOUGH2 with open-source FEM software Code_Aster	THM	caprock damage, fault reactivation, large-scale mechanical analysis	^139^
	TOUGH2-RDCA	coupling TOUGH2 with a discontinuous cellular automaton model to simulate fluid-driven fracture interactions	HM (fracture propagation)	caprock integrity, mechanisms of injection-induced multiple fracture initiation and propagation	^204^
CMG series	GEM	advanced compositional simulator with built-in fluid–solid coupling functionality	HM, THM	industrial-scale storage capacity assessment, injection strategy optimization, and geomechanical risk analysis	^205,206^
	STARS	thermal recovery, chemical reaction, and geomechanics simulator	THC, HM	storage considering chemical reactions, salt precipitation effects, mineral dissolution compensation	^207,208^
	IMEX	black-oil simulator for rapid simulation of CO_2_-brine two-phase flow	H	rapid two-phase flow simulation, capillary heterogeneity effects	^209^
ECLIPSE series	ECLIPSE 300	compositional simulator, accurately simulates CO_2_ phase behavior	H	storage potential screening, long-term plume migration prediction, coal seam storage feasibility	^210^
	ECLIPSE	industry-standard oil and gas reservoir simulation software	H	detailed plume migration, impact of thin shale barriers, evolution of storage mechanisms	^211,212^
	ECLIPSE-ABAQUS	coupling of ECLIPSE with FEM software Abaqus	THMC	assessing the interplay between CO_2_ injection and activities like coal mining	^213^
	ECLIPSE-OpenGeoSys	coupling of ECLIPSE with the multiphysics open-source platform OGS	HMC	integrates the advantages of industrial standards and research platforms for fully coupled simulation	^214^
STOMP series	STOMP-CO_2_	simulates subsurface multiphase flow, heat transport, and CO_2_ migration	THC	leakage risk assessment, vadose zone transport, basalt mineralization storage	^215,216^
	STOMP-COMP	simulates coinjection of CO_2_ with impurity gases (e.g., H_2_S)	THC	dissolution and mineral trapping of CO_2_ with impurities	^217^
	STOMP-ABAQUS	coupling of STOMP with Abaqus	THM	fully coupled THM analysis of injection process, fracture risk	^218^
	eSTOMP-RBSM	coupling of STOMP with RBSM	HM	influence of fault zones on pressure accumulation and surface uplift	^219^
OpenGeoSys (OGS)	Core platform	open-source multiphysics simulation platform, based on FEM/FVM	THMC	reactive transport, mechanical deformation, multifield coupling mechanisms, leakage risk	^163^
	OGS-ChemApp	coupling of OGS with geochemical software ChemApp	HC, THC	accurately simulates complex geochemical reaction pathways, long-term kinetics	^220^
FLAC3D series	FLAC3D-RS-CO2	coupling scheme of a specific multiphase flow simulator with FLAC3D	HM	influence of injection schemes on fault activation and induced seismicity	^221^
	FLAC3D-MUFITS	coupling of multiphase flow simulator MUFITS with FLAC3D	HM	geomechanical risk in faulted reservoirs, fault deformation, and near-wellbore fracturing	^222^
MRST series	MRST (and coupling schemes)	MATLAB reservoir simulation toolbox	H (extendable to HC, HM)	rapid prototyping, history matching, plume migration, global sensitivity analysis	^223,224^
	MRST-FLAC3D	coupling of MRST with FLAC3D	HM	parameter sensitivity and geomechanical risk assessment	^15,225^
	MRST-HYD	extension module of MRST for nonisothermal reactive multicomponent multiphase flow	THC	deep-sea CO_2_ storage, hydrate formation and dissolution	^226^
PyLith series	PyLith-FlowSim	coupling framework for mechanical software PyLith and a flow simulator	HM	influence of CO_2_ solubility on fault leakage rate and poroelastic instability	^227^
	PyLith-MRST	coupling of the mechanical software PyLith with the flow toolbox MRST	HM	control of low-permeability faults on CO_2_ migration and induced seismicity risk	^228^
NUFT series	NUFT (and coupling schemes)	multiphase flow and heat transport simulator	TH	CO_2_ plume geothermal systems, injectivity and heat extraction efficiency assessment	^229,230^
	NUFT-RSQSim	coupling of NUFT with earthquake simulator RSQSim	HM (induced seismicity)	control of induced seismicity through active pressure management	^231^
COMSOL Multiphysics	core platform	integrated multiphysics simulation software based on FEM	fully coupled THMC	fine-scale simulation near wellbore, parameter sensitivity analysis, and conceptual model validation	^232,233^
other commercial and open-source tools	PFLOTRAN	open-source, massively parallel reactive transport simulator	THC	long-term geochemical evolution, large-scale high-resolution simulation, uncertainty quantification	^181,234^
	PHREEQC and PhreeqcRM	geochemical computation software (batch and reactive transport modules)	C (often used as a coupling component)	batch geochemical calculations, reaction path modeling, pore-scale reactive transport	^235,236^
	CrunchFlow	reactive transport simulation software	HC	reservoir property evolution, cement-rock interactions, mineral precipitation effects	^237,238^
	CooresFlow	reactive transport simulator	HC	aquifer CO_2_ leakage experiment simulation, leakage tracer studies	^239,240^
	DMflow	multicomponent nonisothermal seepage simulator	TH (multicomponent)	prediction of gas phase behavior, temperature distribution, and multicomponent effects during CO_2_ injection	^241^
	OPM Flow	open-source oil and gas reservoir simulator	H	long-term storage performance assessment, parameter uncertainty, and global sensitivity analysis	^242^
	DuMu^x^	C++ based open-source multiphase flow simulation framework	TH	CO_2_ migration in fractured reservoirs, enhanced geothermal system simulation	^182^
	LandSim	storage site performance assessment software	H	storage strategy development, injection parameter optimization, plume migration control	^243^
	openform	commercial oil and gas reservoir simulation software	H	two-phase flow characteristics in heterogeneous reservoirs, CO_2_ migration patterns	^244^
	abaqus (and user subroutines)	general-purpose finite element analysis software	HM, THM	caprock stability, wellbore integrity, and detailed reservoir deformation analysis	^245,246^
	GEOSIM	HM coupling simulation software	HM	caprock fracturing risk, effect of injection temperature on fracture propagation	^247^
	DARSim	multiphase flow simulator	H	comparison of plume migration characteristics and storage mechanisms for different gases (CO_2_, H_2_)	^248^
	COMPASS	THMC dual-porosity model simulator	THMC	horizontal well injection scheme optimization, control of diffusion range	^18^
	PetroMod	basin modeling software	TH (long-term evolution)	storage feasibility, long-term assessment of fault leakage risk	^249^
	GEOS	multiscale multiphysics open-source simulator	THM (fully coupled)	fluid–solid coupling simulation near wellbore, wellbore stability and leakage risk assessment	^250^
	IPARS	parallel multiphase compositional reservoir simulator	H (multicomponent)	solute transport and multiphase compositional effect uncertainty analysis, model verification	^251^
	Geochemist’s Workbench (GWB)	geochemical modeling software	C	mineral reactivity assessment, prediction of long-term mineral trapping potential	^252^

Among the many platforms, the TOUGH2 series occupies a dominant position in CGS coupled simulation due to its open architecture, extensive fluid and property modules, and active community development.^164–166^ Its core advantage lies in the flexible combination of modules: TOUGHREACT incorporates comprehensive geochemical reaction pathways, significantly enhancing the predictive capability for CO_2_ mineral trapping potential and reservoir-caprock chemical alteration, for instance, successfully simulating mineral dissolution–precipitation sequences induced by impure CO_2_ injection.^160,167,168^ TOUGH2-FLAC3D represents a classic scheme for HM coupling, achieving fully dynamic two-way feedback between fluid flow and rock mass deformation through iterative solving.^169,170^ As shown in Figure 12, this coupling mechanism operates within each time step: first, TOUGH2 solves the pressure field and transfers it to FLAC3D; FLAC3D then computes the mechanical response and deformation based on the effective stress principle; and finally, the updated parameters, such as porosity and permeability, are fed back to TOUGH2 to advance the calculation to the next time step. This mechanism is essential for modeling geomechanical issues, such as fault reactivation driven by injection, caprock shear failure, and surface uplift.

**Figure 12:**
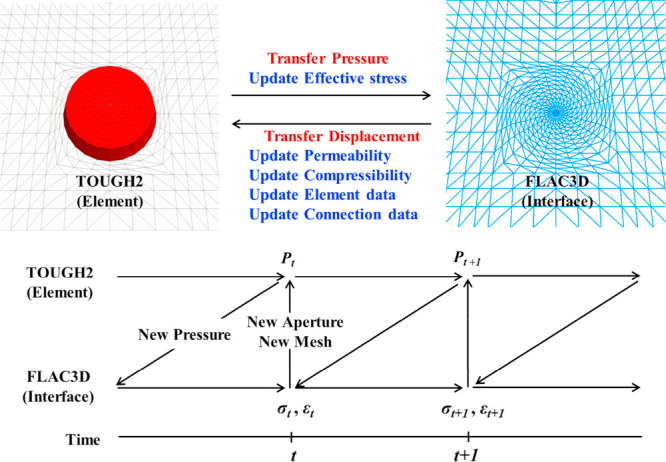
Data exchange mechanism between TOUGH2 volume elements and FLAC3D interface elements. This figure was reproduced with permission from ref 185. Copyright 2020 Elsevier.

Along with the TOUGH series, CGS engineers also use commercial reservoir simulators like CMG and ECLIPSE a lot.^171,172^ CMG’s GEM compositional simulator has advanced HM coupling features that make it easy to simulate changes in reservoir pressure caused by CO_2_ injection and how these changes affect the mechanical properties of rocks. This helps with capacity evaluation and injection optimization for large-scale storage projects.^173^ The ECLIPSE 300 compositional simulator accurately depicts the CO_2_ phase behavior, making it well-suited for simulating the complex phase behavior of CO_2_–CH_4_ mixtures in depleted gas reservoirs or coal seams. This makes it a useful tool for analyzing ways to improve energy recovery and storage strategies.^162^ Furthermore, the STOMP series (e.g., STOMP-CO_2_ and STOMP-COMP) developed by PNNL focuses on multiphase flow, heat transport, and chemical reactions of impure CO_2_ in the vadose zone and aquifers, offering unique advantages for leakage scenario simulation and long-term geochemical evolution prediction.^174,175^

Professional and open-source software further expands the research methodologies for CGS simulation. COMSOL Multiphysics, as a general-purpose FEM-based multiphysics platform, is suitable for high-resolution simulation of complex geometric regions near the wellbore and strongly coupled processes, often used for validating newly proposed THMC conceptual models or conducting parameter sensitivity analysis.^176^ PHREEQC and its reactive transport module PhreeqcRM have become the de facto standard for geochemical simulation, serving both as a standalone tool to assess mineral reactivity and long-term storage potential, and as a chemical engine embedded within other flow simulators to enable detailed characterization of pore-scale reactive transport processes.^177–179^ Open-source platforms such as MRST, PFLOTRAN, and DuMux, by virtue of their transparency, extensibility, and excellent parallel capabilities, are becoming significant enablers for conducting large-scale, high-resolution, fully coupled THMC simulations and uncertainty quantification research.^180–182^ These diverse tools collectively form a multilevel, functionally complementary numerical simulation ecosystem for CGS, continuously propelling the science and technology in this field to deeper levels of development.^183,184^

### 3.4 Perspectives and Prospects

Current research on THMC coupling methods still faces multiple challenges. At the coupling strategy level, although the fully coupled method can accurately describe multifield interactions, its high computational cost restricts its application in field-scale and long-term simulations.^137^ Sequential coupling, as a compromise, has its convergence and stability significantly influenced by the iterative algorithm and time step size, making it prone to introducing substantial errors in strongly nonlinear transient processes (e.g., near-wellbore injection and initial fault reactivation).^127^ Defining acceptable convergence and stability criteria is crucial for ensuring the reliability of sequential coupling, a two-way iterative scheme; however, consistent and explicit standards for these criteria are currently lacking. The convergence criterion must prioritize the iterative consensus of the interconnected variables. For important partners in THMC coupling, such as stress-permeability, temperature-reaction rate, and deformation-porosity, the difference between two iterations must be less than 5%. At the same time, the residuals from the various governing equations of the fields (such as flow, heat conduction, and mechanical equilibrium) need to be limited so that they do not cause localized convergence or poor coordination across fields. The stability criterion must take into account how nonlinear the system is and how it is broken down into time periods. This means that the time step size must be adjusted dynamically based on how quickly each physical process reacts. For example, smaller steps should be used for flow and chemical fields that change quickly, whereas greater steps should be used for deformation fields that change slowly. To stop numerical divergence, the number of iterations for each time step must be restricted.

Also, the order of the iterations needs to be changed depending on how sensitive the interfield coupling is (for example, by giving updates to the flow-thermal fields more weight than updates to the mechanical-chemical fields) to cut down on numerical oscillations. The lack of clarity in these criteria causes a lot of differences in the choices of parameters used in the study. Consequently, comparing results from several simulations is challenging, resulting in discrepancies between model predictions and the real field observations. This significantly undermines the practical reliability of the sequential coupling method in engineering applications. In the field of numerical methods, continuum approaches (e.g., FEM, FVM) struggle with discontinuous media and scaling problems, whereas discrete methods (e.g., DFN, DEM) have trouble growing to engineering scales because of limits on computing power.^145,146^ Furthermore, critical coupling characteristics, such as stress-dependent permeability and reactive surface area, are difficult to accurately obtain and quantify on the field scale, significantly increasing the uncertainty in model validation and prediction. The combination of these traits limits the ability of THMC models to make quantitative predictions and help with decision-making in real storage projects. Figure 13 systematically outlines the principal challenges and prospective opportunities related to current THMC coupling approaches.

**Figure 13:**
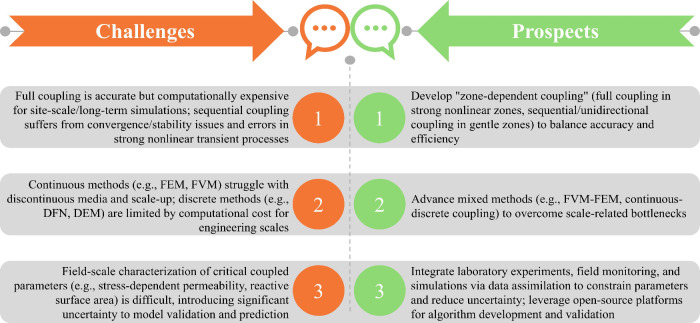
Present challenges and future advancements in THMC coupling techniques.

Future research should focus on developing sophisticated hybrid coupling methods and high-performance computing frameworks to enhance the balance between simulation accuracy and efficiency. Explicit instructions entail the examination of “zonal coupling” strategies that allocate coupling methods dynamically based on the spatiotemporal characteristics of physical processes—employing full coupling in highly nonlinear regions and sequential or one-way coupling in areas with gradual variations. To get beyond the scale constraint, one important method is to improve multiscale hybrid modeling (for example, FVM-FEM or continuum-discrete coupling). To combine laboratory experiments, field monitoring, and numerical simulations, data assimilation techniques must be used at the same time. This successfully limits model parameters and reduces forecast uncertainty.^144^ Open-source platforms like OGS and PFLOTRAN are the best places to design, test, and compare the algorithms mentioned above because they are open and can be changed easily.^183^ Through these activities, THMC coupling approaches are expected to evolve from mechanistic research tools to practical engineering design and risk management platforms, providing crucial simulation support for the secure large-scale implementation of CGS.

## 4 THMC Coupling Applications

### 4.1 Site Screening

Site screening is an important part of a CGS project since it involves finding suitable places with a lot of storage space, long-term stability, and little damage to the environment.^24,25^ Conventional screening mainly focuses on large-scale geological features, such as structural closure, reservoir-seal pairings, and the initial pressure regime.^253^ As our understanding of THMC coupling behavior grows, the screening process changes from a static geological evaluation to a dynamic process assessment. This means that we need to carefully look at how the reservoir and caprock change over time due to multifield coupling during both the injection and postinjection phases, as well as how these changes affect long-term storage safety and effectiveness.^254^ This change requires more precise and complete site characterization data. This means that information from different fields needs to be combined to create a vital parameter system that appropriately reflects THMC activities.^255^ The primary objective of THMC coupling in this context is to accurately quantify the impact of multiphysics interactions on the reservoir’s effective storage capacity and the temporal dynamics of caprock-fault system stability, aiming to establish essential suitability thresholds for CCUS implementation in various geological settings.

THMC coupling analysis provides a more comprehensive scientific framework for site appraisal, including both reservoir potential assessment and risk management.^256,257^ When assessing capacity, it is important to measure the synergistic and antagonistic effects of THMC processes. For example, mineral reactions can improve mineral trapping but may make porosity and injectivity worse. Changes in effective stress can change fracture aperture and reservoir compressibility, which changes dynamic capacity. Research indicates that while high porosity (>10%) facilitates injection, it readily leads to the formation of preferential flow paths, weakening mineral reaction efficiency; whereas reservoirs rich in reactive minerals like anorthite (>10 wt %) significantly enhance long-term mineralization potential.^258,259^ In terms of risk assessment, THMC analysis focuses on caprock integrity, fault reactivation, and leakage risk, with key indicators including caprock breakthrough pressure, fault friction coefficient, and the chemomechanical degradation degree of wellbore cement. Numerical simulation is a core tool for quantifying these interactions; for instance, coupled flow-solid-chemical simulation can assess the long-term impact of CO_2_-rock interactions on reservoir strength.^260^ While risk models integrating THMC constraints (e.g., NRAP-IAM-CS) can systematically predict the probability of environmental impacts under different leakage scenarios.^261,262^ In the future, establishing standardized screening procedures and rapid assessment tools based on THMC coupling will be key to advancing CCUS from demonstration to large-scale commercial application.

### 4.2 Wellbore Evaluation

The wellbore, as the sole artificial conduit connecting the surface to the reservoir, is the weakest potential leakage point in CGS, and its integrity is directly related to the long-term safety of the entire project.^263–265^ Throughout the entire lifecycle of CO_2_ injection, equilibration, and long-term storage, the wellbore cement, casing, and their bonding interfaces with the surrounding rock continuously undergo complex THMC coupling effects, potentially triggering a series of degradation phenomena such as cement carbonation, steel corrosion, and interfacial debonding, significantly compromising their sealing and mechanical properties.^266^ Traditional single-field assessment methods struggle to accurately predict such multiphysics synergistic degradation processes, urgently necessitating the establishment of an integrity evaluation framework capable of characterizing THMC coupling effects. Dalla Vecchia et al.^267^ systematically revealed the degradation mechanism of cement-casing systems with interfacial defects in CO_2_-enriched brine through experiments; the kinetic model they proposed clearly delineates the sequential chemical reactions (e.g., precipitation sequences of CaCO_3_, FexCayCO_3_, FeCO_3_) and accompanying ion migration processes occurring at the material interfaces (Figure 14). In this scenario, the core focus of THMC coupling is on the multifield synergistic degradation mechanisms involving CO_2_-brine-wellbore materials (cement/casing) and the coupled effects of transient thermal-pressure stresses and chemical interactions in the near-wellbore zone, aiming to accurately characterize the long-term evolution of wellbore sealing performance and provide a targeted analytical framework for integrity assessment.

**Figure 14:**
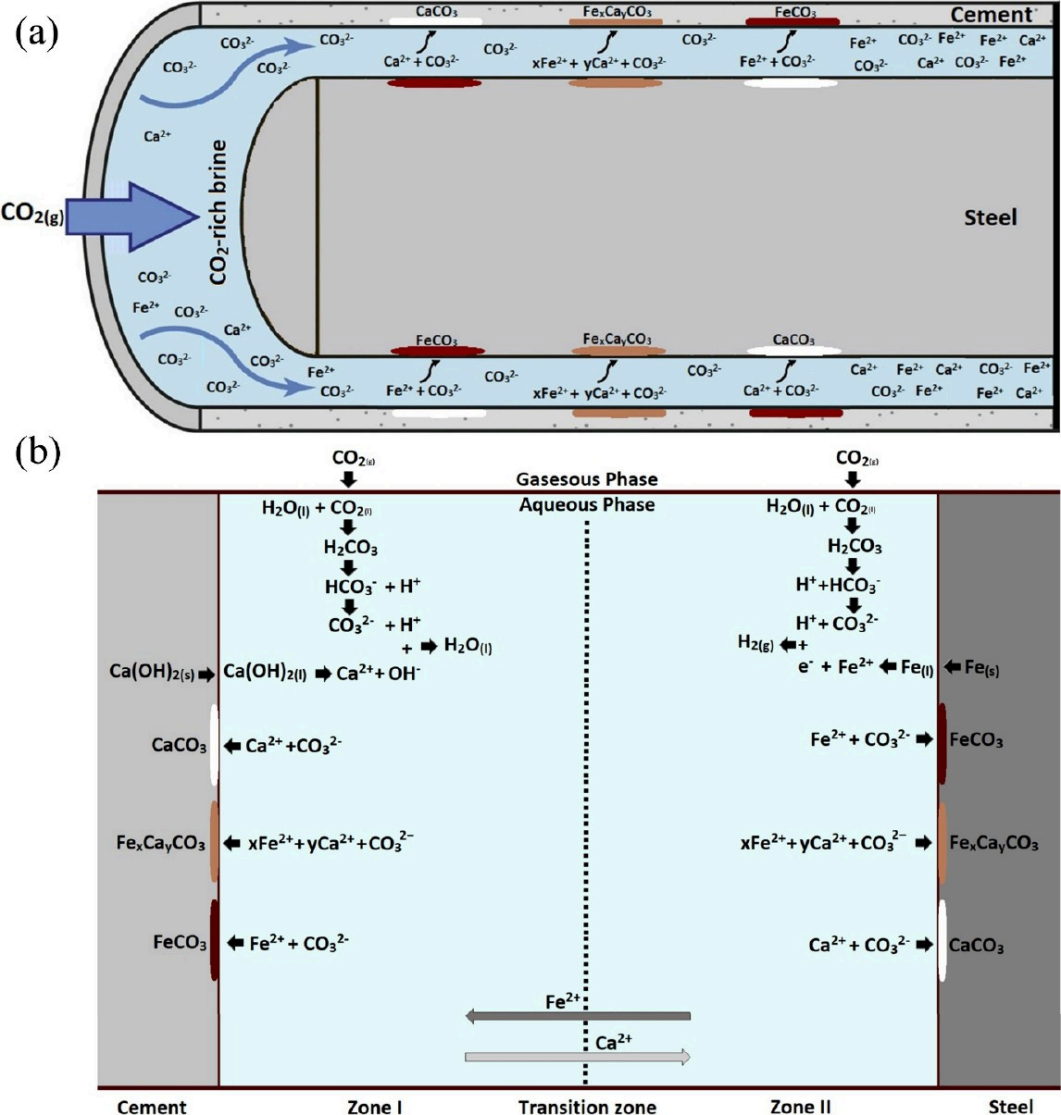
(a) Flowing brine-rich environment coupling kinetic processes and material exchange within the cement-casing-CO_2_ system; (b) chemical reaction mechanisms in the cement layer (Zone I) and the N80 corrosion zone (Zone II) of the cement-casing system. This figure was reproduced with permission from ref 267. Copyright 2020 Elsevier.

The application of THMC coupling analysis in wellbore integrity assessment is primarily reflected in the refined prediction of material degradation mechanisms, near-wellbore transient processes, and long-term evolutionary behavior. At the HMC coupling level, it is necessary to quantitatively evaluate the kinetic processes of CO_2_-brine-cement/steel reactions and their long-term effects on material mechanical properties; for instance, calcite precipitation may induce a self-healing effect via pore clogging, while volume changes might also trigger the initiation and propagation of microfractures.^268^ At the TH coupling level, the phase behavior of injected CO_2_, temperature distribution, and thermal stress are key to integrity assessment; thermal shock from low-temperature injection may cause brittle fracture of the cement sheath, while multiphase flow under nonisothermal conditions governs leakage rates and extent.^269,270^ Numerical simulation tools, such as the wellbore-reservoir coupled simulator T2Well/ECO2N, have been successfully used to reveal complex near-wellbore flow phenomena like periodic eruptions and to accurately characterize the differential migration of thermal and saturation fronts.^26,193^ In the future, efforts should focus on developing fully coupled numerical platforms that integrate microscopic material degradation models, fracture propagation criteria, and macroscopic multiphase flow dynamics. Combined with real-time monitoring data, such as from fiber optic sensing, these platforms should form the basis for a dynamic wellbore risk assessment and early warning system grounded in THMC coupling, providing a scientific basis and technical support for the safe design and operation of CGS projects.

### 4.3 Storage Mechanisms

The long-term safety and effectiveness of CGS depend on the synergistic action and spatiotemporal evolution of multiple storage mechanisms.^271,272^ The primary mechanisms include structural trapping, residual trapping, solubility trapping, and mineral trapping. They dominate the CO_2_ immobilization process at different time scales, collectively forming a dynamic multibarrier storage system.^273^ Structural trapping relies on the physical confinement offered by the caprock and is most effective during the initial storage phase. Residual trapping keeps CO_2_ in the pores using capillary forces. Solubility trapping involves dissolving CO_2_ in formation water. Mineral trapping, on the other hand, allows for near-permanent storage through water-rock interactions that create stable carbonates.^274–276^ To predict how well the storage system will work in the long run, it is important to understand how these systems work together and how they respond to changes in the reservoir.^277–279^ Figure 15 shows the multiphase distribution next to the injection well. It clearly shows the dried zone, two-phase zone, and single-phase zone arranged in that order outward from the wellbore. It also shows the spatial distribution and dominance of the four trapping mechanisms in each zone. Table 6 systematically compares the multidimensional characteristics of CGS trapping mechanisms. The central objective of THMC coupling within this context is to elucidate how multiphysics interactions dynamically govern the efficacy of the four primary trapping mechanisms (structural, residual, solubility, and mineral) and to unravel their intermechanism synergy, competition, and spatiotemporal evolution. This approach furnishes an accurate, multifield coupled framework for assessing the long-term CGS performance.

**Figure 15:**
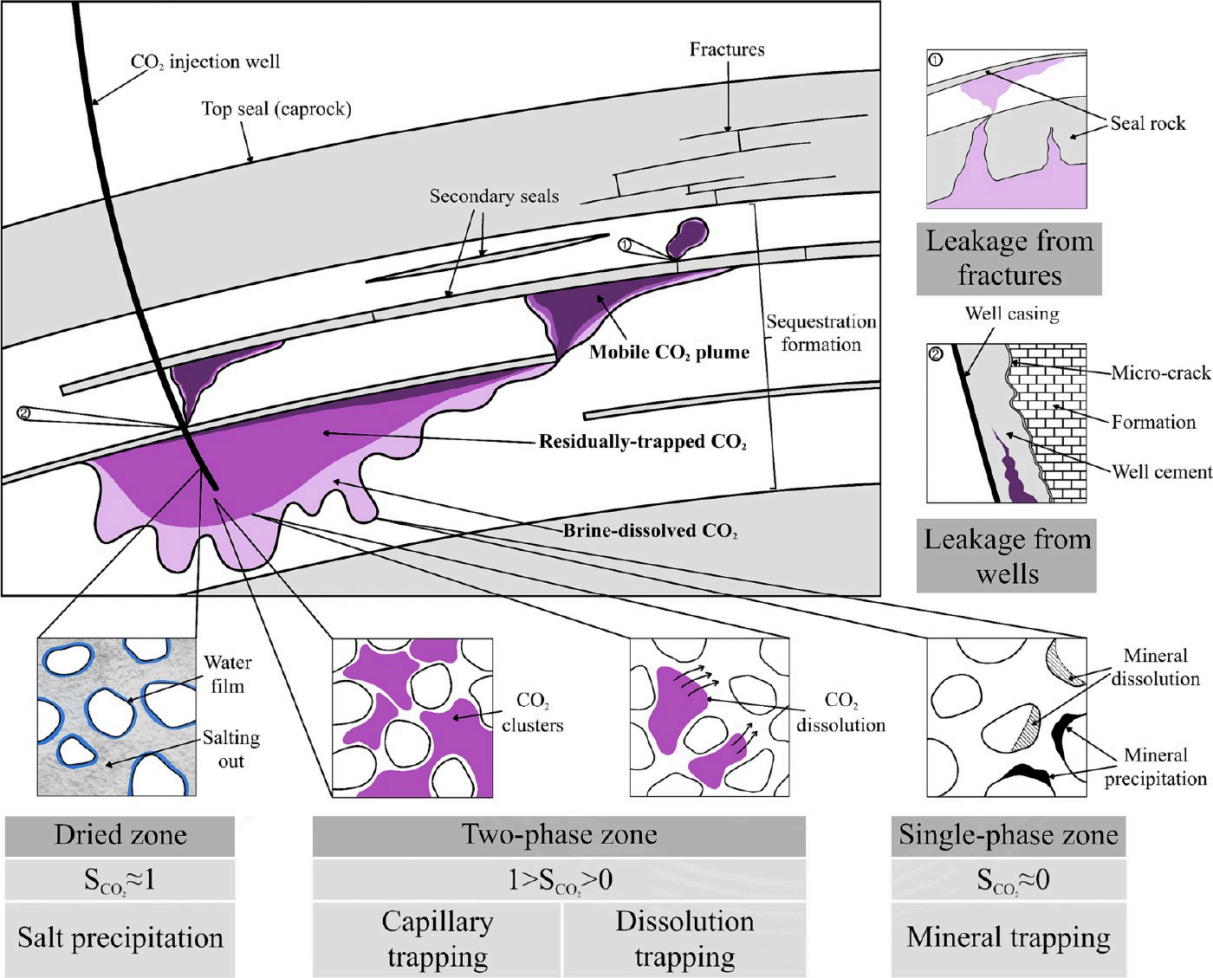
Schematic diagram of phase distribution, geological architecture, four primary trapping mechanisms, and key physicochemical processes in a saline aquifer near an injection well: includes dried zone, two-phase zone, and single-phase zone (characterized by CO_2_ saturation profile from the injection well to the far field). This figure was reproduced with permission from ref 290. Copyright 2023 Elsevier.

**Table 6:** Multi-Dimensional Comparative Analysis of CGS Trapping Mechanisms

mechanism dimension	structural trapping	residual trapping	solubility trapping	mineral trapping
dominant process and principle	accumulation of supercritical CO_2_ at structural highs beneath low-permeability caprock^292^	CO_2_ is snap-off and immobilized in pores by pore-throat capillary forces^293^	CO_2_ dissolves into formation water, forming carbonic acid^294^	CO_2_ reacts with reactive minerals to form carbonates^295^
time scale	instantaneous to decades	injection period to centuries	decades to millennia	centuries to tens of thousands of years
capacity contribution characteristics	dominant early stage, contribution can reach 79%^296^	medium to high, 42% higher in water-wet systems than in CO_2_-wet systems^297^	medium, increased temperature can enhance the dissolution amount^294^	dependent on mineral composition and reaction kinetics^295^
long-term stability	dependent on caprock integrity, leakage risk exists	relatively stable, pressure fluctuations may cause remobilization^293^	stable, no buoyancy after dissolution	highly stable, nearly permanent
key controlling factors	structural configuration, caprock integrity, and faults^298^	pore structure, wettability, permeability hysteresis^299^	convective mixing, salinity, pH, pressure^300,301^	reactive mineral content, temperature, pH, kinetics^258^
THMC coupling effects	HM coupling: Injection pressure affects fault/caprock stability^302^	HC coupling: Dynamic wettability affects residual saturation^303^	THC coupling: Temperature and convection jointly control dissolution rate	THMC coupling: Reactions alter pore structure and mechanical properties
simulation challenges and frontiers	accurate characterization of large-scale structures and fault systems	quantification of relative permeability and capillary pressure hysteresis effects	quantification of large-scale convective mixing intensity	prediction of long-term reaction kinetics and upscaling

THMC multifield coupling processes profoundly influence the efficiency and long-term stability of each storage mechanism, which has been deeply revealed through recent simulations and experiments.^280,281^ Regarding THM coupling, Zhong et al.^282^ studied wellbore-reservoir coupling in CO_2_-based enhanced geothermal systems (EGS) and found that the strong Joule-Thomson effect causes a sharp temperature drop in the wellbore, directly affecting the CO_2_ phase behavior and mobility in the near-wellbore zone. Regarding HC coupling, Wang et al.^283^ revealed through full-cycle simulation that residual trapping is dominated by advective transport during the injection period, while solubility trapping is alternately controlled by advection and gravitational convection. Rezk and Ibrahim^284^ further found that geological heterogeneity significantly affects the proportions of structural, solubility, and residual trapping by altering flow paths. Wang et al.^285^ simulated and showed that HCO_3_^–^-assisted oxidative dissolution of UO_2_ is a potential uranium mobilization mechanism in uranium-bearing mineral formations, but even under the worst-case scenario, it does not significantly migrate upward to shallow aquifers; this process involves the impact of chemical reactions on contaminant transport. Furthermore, studies by Ubillus et al.^286^ and Khoramian et al.^287^ emphasized the key controlling role of hydraulic characteristics such as reservoir heterogeneity and relative permeability hysteresis effects on residual trapping efficiency, respectively. These studies demonstrate that THMC coupling effects are interwoven and collectively govern the fate of the CO_2_.

In summary, the understanding of CGS mechanisms needs to shift from a static, single-perspective view to a systematic perspective of dynamic multifield coupling.^288^ Future research needs to develop numerical models capable of accurately describing the fully coupled THMC processes, focusing on addressing challenges such as long-term reaction kinetics prediction, multiscale heterogeneity characterization, and quantification of relative permeability hysteresis effects.^289,290^ A feasible path involves integrating a mechanistic understanding from laboratory experiments with field monitoring data to constrain and calibrate complex models through data assimilation techniques. Simultaneously, engineering intervention measures can be explored, such as the artificial silica gel barrier proposed by Cossins et al.,^291^ to actively guide and optimize the contributions of different storage mechanisms. Only through a systematic approach that combines mechanistic research, simulation prediction, and engineering management can an accurate assessment and active control of the long-term safety of CGS be ultimately achieved, providing a scientific basis for the large-scale application of carbon storage technology.

### 4.4 Caprock Evaluation

Caprock integrity evaluation is central to the safety of CGS, aiming to study the ability of low-permeability caprocks to resist fluid migration and maintain mechanical stability under long-term service conditions.^304^ The caprock experiences intricate THMC coupling effects throughout the entire lifecycle of CO_2_ injection and storage. These effects include injection pressure and buoyancy exceeding its capillary entry pressure, effective stress changes leading to shear slip or tensile fracturing, CO_2_-brine-rock reactions modifying mineral composition and pore structure, and temperature fluctuations resulting in the generation and evolution of thermal stresses.^305–307^ These interrelated processes collectively dictate the long-term sealing efficacy of the caprock, rendering conventional single-physics evaluation techniques insufficient for engineering requirements.^308^ Figure 16 illustrates the dynamic process of CO_2_ invading a low-permeability caprock under capillary control, distinguishing between two migration mechanisms: free-phase seepage and dissolved-phase diffusion, providing a key perspective for understanding the physical essence of caprock sealing failure. In this scenario, the core focus of THMC coupling is on the dynamic control mechanisms of pressure–temperature-chemical interactions on caprock sealing performance and the quantification of the caprock mechanical instability threshold induced by multifield coupling, aiming to identify key suitability indicators for different caprock structures, mineral compositions, and reservoir conditions.

**Figure 16:**
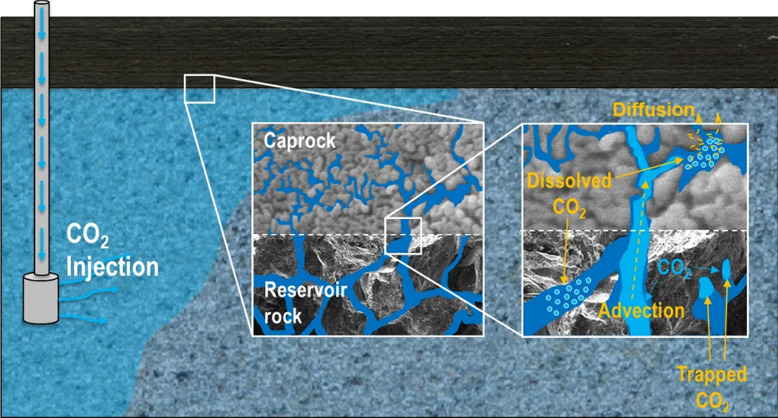
Diagram showing how CO_2_ gets into a caprock with a low permeability. This figure was reproduced with permission from ref 315. Copyright 2022 Springer Nature.

THMC coupling analysis demonstrates significant value in caprock assessment.^309^ Taghizadeh et al.^310^ employed a 3D geomechanical model and the Mohr-Coulomb criterion to quantify the critical CO_2_ injection rate, indicating that the horizontal/vertical stress ratio is a key mechanical factor controlling integrity. Gor et al.,^311^ through thermo-poro-mechanical coupling simulation, revealed the TM mechanisms by which low-temperature CO_2_ injection induces tensile or shear failure in the caprock. Punnam et al.^312^ applied multiphase, multicomponent reactive transport simulation and found that stair-stepped caprock configurations exhibit optimal leakage prevention performance. Alsayah and Rigby^313^ utilized an HMC coupling approach to reveal the geo-mechanical feedback mechanisms by which complex caprock structures lead to CO_2_ leakage. Aminaho et al.^314^ found that injecting impure CO_2_ (with SO_2_/H_2_S) at the same time has a big effect on the brittleness index and permeability of caprock. This shows how important HMC coupling is. Figure 17 systematically clarifies the dual mechanism of thermal stress: it can enhance caprock stability during initial injection, while extended cooling may create a tensile stress state, increasing the likelihood of fractures; this phenomenon is collectively influenced by factors such as the reservoir-caprock stiffness ratio, thermal expansion coefficient, and Biot’s coefficient.

**Figure 17:**
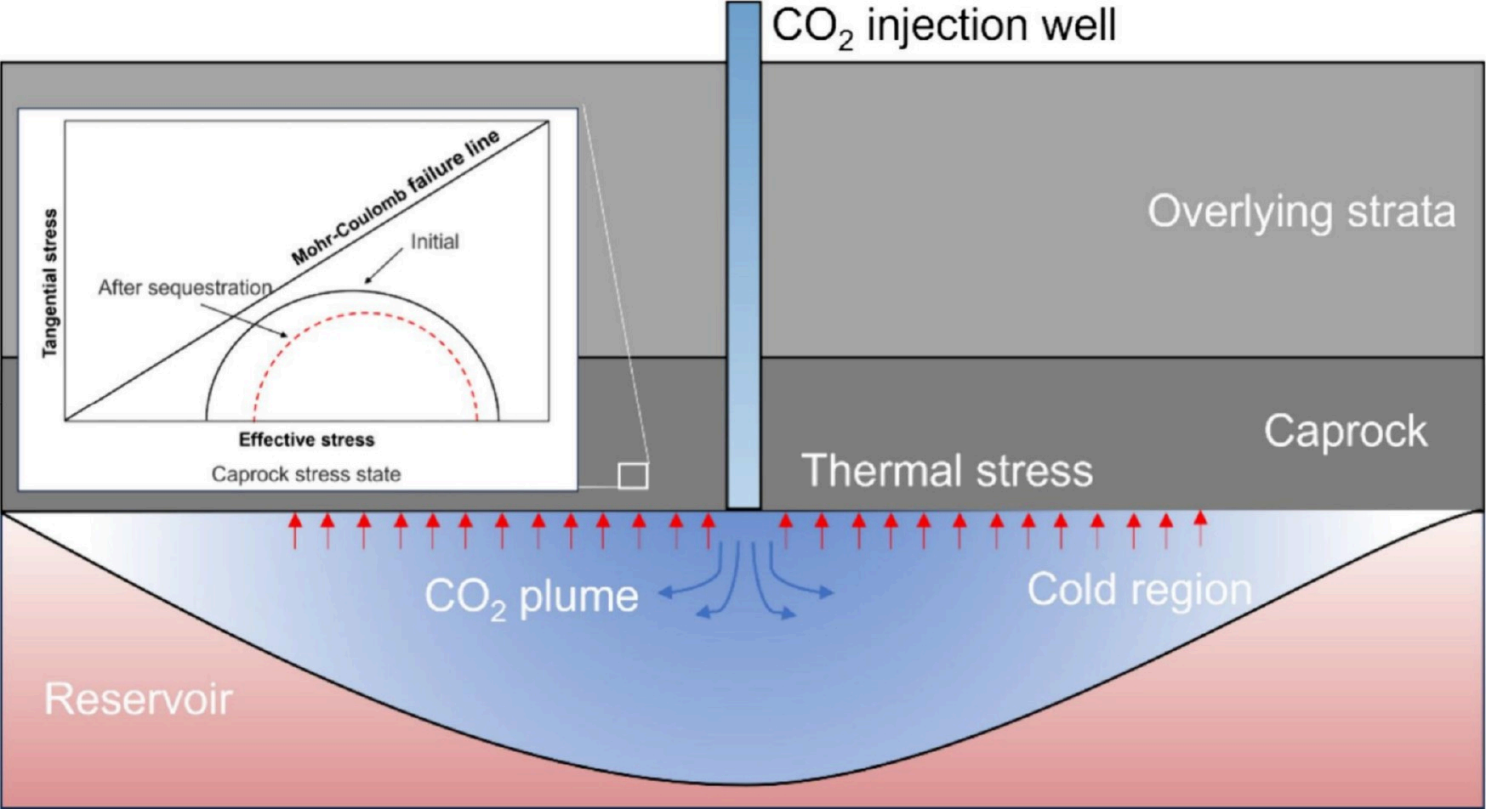
Diagram showing how the passage of CO_2_ causes thermal stress that affects the stability of caprock. This figure was reproduced with permission from ref 36. Copyright 2024 Elsevier.

In conclusion, the assessment of caprock integrity has evolved from a traditional mechanical analysis to a thorough evaluation that requires the incorporation of complete THMC coupling. Future efforts should promote the development of more advanced multifield coupled numerical models to precisely represent the long-term evolution of caprocks exposed to thermal shock, chemical erosion, and pressure fluctuations. A feasible strategy involves the integration of laboratory mechanistic data (e.g., capillary entry pressure and mineral reaction kinetics) with site models and real-time monitoring information (e.g., microseismicity and surface deformation) to create a dynamic early warning system through data assimilation. In engineering, sites with good morphology (like stairs), clear capillary heterogeneity, and the right mineral composition should be given priority. Also, injection tactics (such as controlling temperature and rate) need to be improved to make the several caprock processes work better together, which will keep CGS safe in the long run.

### 4.5 Fault Evaluation

For the safety of CGS, it is important to perform a fault assessment. This mainly means being able to accurately forecast how fault systems would work over time. They can either be leakage channels or sealing barriers.^316–318^ Fault zones have complex structures and are very sensitive to the impacts of the THMC coupling. Pressure changes caused by injection can lower the effective normal stress, which can cause faults to reactivate. Stress redistribution can lead to shear slip or tensile fracturing. CO_2_-fluid-mineral interactions can change pore structure and permeability by dissolving and precipitating minerals. Changes in temperature create thermal stresses that affect fault frictional properties and stability.^319–321^ The nonlinear connections among these processes make it very hard to predict how faults would behave. This means that we need to quickly come up with new analytical tools that go beyond traditional single-physics approaches.^322^ Figure 18 systematically shows the relationship between the global distribution of CO_2_ storage projects and active faults, revealing the widespread influence of fault structures on storage site selection and highlighting the necessity of fault assessment during the project planning phase. In this scenario, the core focus of THMC coupling is on the critical conditions for fault reactivation induced by stress-pressure-chemical coupling and the dynamic evolution of fault sealing capacity (self-sealing vs leakage pathways), aiming to define the stability suitability thresholds under different fault geometries, mineral compositions, and reservoir conditions.

**Figure 18:**
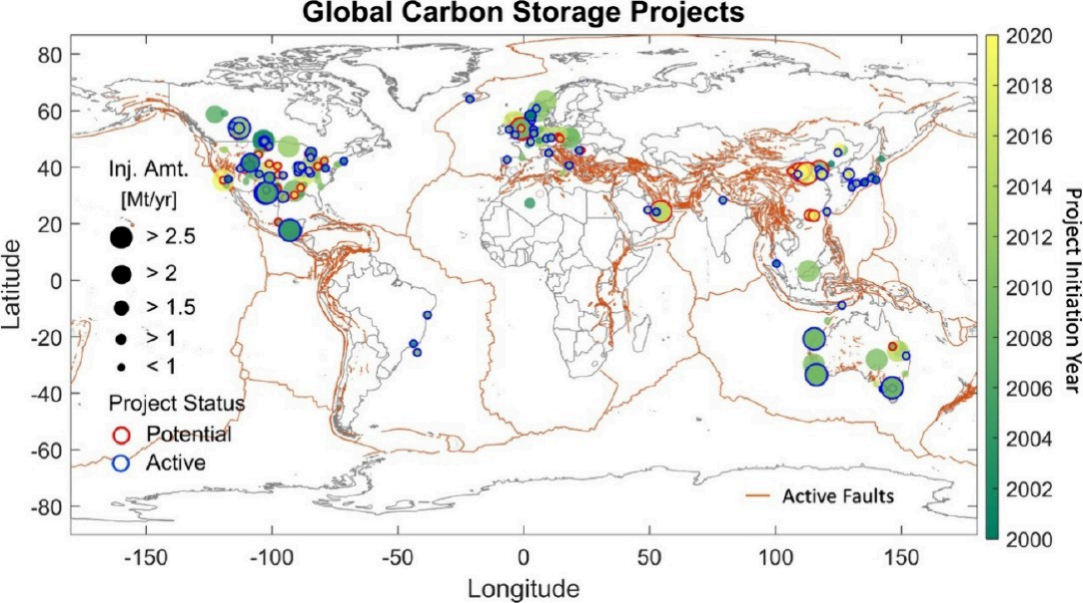
Global map of the CO_2_ storage projects: Active fault lines are marked in orange; includes commercial-scale CO_2_ storage projects (≥2.5 Mt/year) planned or initiated since 2008, color bar represents project start year, circle size reflects targeted formation CO_2_ injection rate; “Potential” refers to projects in planning (including site characterization, plant design, and/or capture progress), “Active” refers to projects in injection or postinjection monitoring. This figure was reproduced with permission from ref 331. Copyright 2022 American Geophysical Union.

THMC coupling analysis provides an effective tool for understanding fault behavior.^323–325^ Patil and McPherson,^326^ using a spatiotemporally variant Damköhler numerical framework, revealed the MC mechanism by which shallow limestone faults achieve self-sealing through carbonate precipitation under specific geochemical conditions. Konstantinovskaya et al.,^327^ using a 3D reservoir-geomechanical coupled model, found that a small pore pressure increase (4–6 MPa) can trigger shear slip on high-angle faults. Langet et al.,^328^ based on field monitoring data and Monte Carlo simulation, identified the critical stress mechanism by which pore pressure perturbations can activate far-field faults. Regarding the methodology for fault integrity assessment. Figure 19 systematically explains the potential pathways resulting from fault integrity damage and the four pillars controlling fluid migration along faults—geology, geometry, mechanics, and dynamic behavior—providing a theoretical basis for constructing a comprehensive fault risk assessment framework. This framework emphasizes the need for a systematic assessment across multiple dimensions: the presence and complexity of the fault zone, well-fault geometric relationships, stress state and slip criteria, and fluid dynamic behavior.^329^ Furthermore, Chen et al.,^330^ using a HM coupling model, found that the sealing capacity of reverse and normal faults with dip angles of 60–70 degrees is most sensitive to injection/production strategies.

**Figure 19:**
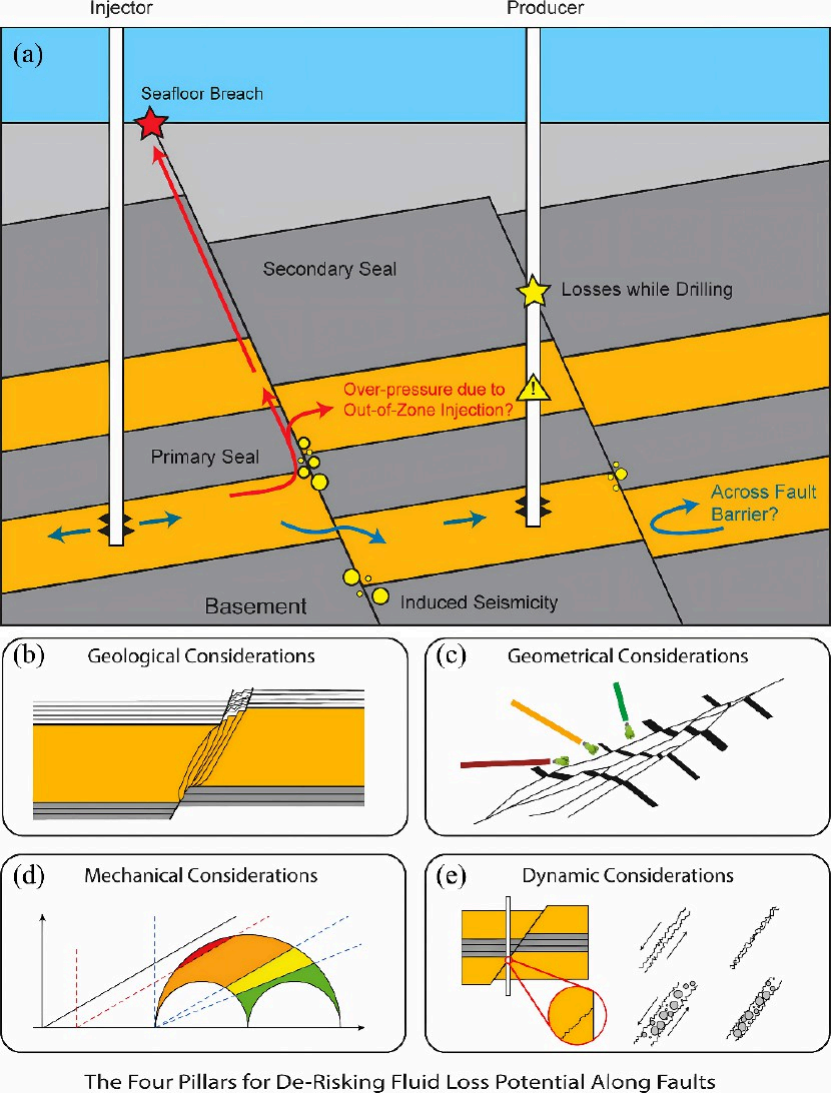
(a) Potential leakage pathways formed due to fault integrity damage in CGS; (b–e) The four pillars of fault integrity. This figure was reproduced with permission from ref 329. Copyright 2024 Elsevier.

In summary, fault assessment has evolved from a traditional mechanical analysis to a THMC system evaluation that requires the integration of geology, geometry, mechanics, and fluid dynamics. Future efforts should make more complex coupled numerical models that can accurately show how things change over time because of things like chemical erosion, changes in pressure, and thermal stress. A feasible strategy involves the integration of laboratory data concerning frictional properties and response kinetics, site-specific geomechanical models, and real-time microseismic monitoring to create a dynamic early warning system for fault reactivation and leakage risk through data assimilation. In engineering practice, sites with favorable orientations, self-sealing potential, and low initial stresses should be prioritized, and strategies such as controlling injection pressure and employing multiwell dispersed injection should be used to minimize fault reactivation risk, ensuring the long-term safety and public acceptance of CO_2_ storage.

### 4.6 Induced Seismicity

The risk of induced seismicity during CGS is a key constraint for the large-scale deployment of projects, with its essence being that the perturbation of the subsurface stress system by injection activities exceeds the fault stability threshold.^332,333^ This process is primarily controlled by THMC coupling effects: injection-induced pore pressure increase directly reduces the effective normal stress on the fault plane; poroelastic effects alter its shear stress state; CO_2_-fluid-mineral reactions influence the frictional properties of fault gouge; and thermal stresses induced by temperature differences also participate in stress redistribution.^334^ These coupled processes collectively regulate the Coulomb stress state on the fault plane, determining its reactivation potential and seismic evolution characteristics.^335^ Figure 20 systematically summarizes three core physical mechanisms for fluid injection-induced seismic activity: the direct effect of pore pressure increases on faults, changes in fault loading conditions, and aseismic slip-triggered seismic slip loading, providing a clear conceptual framework for understanding the multipath origins of induced seismicity. In this scenario, the core focus of THMC coupling is on the synergistic control mechanisms of stress-pressure–temperature-chemical coupling on the fault’s Coulomb stress state and the precise quantification of the nucleation threshold, magnitude, and spatiotemporal evolution patterns of induced seismicity, aiming to identify key indicators for seismic risk management under different fault characteristics and injection conditions.

**Figure 20:**
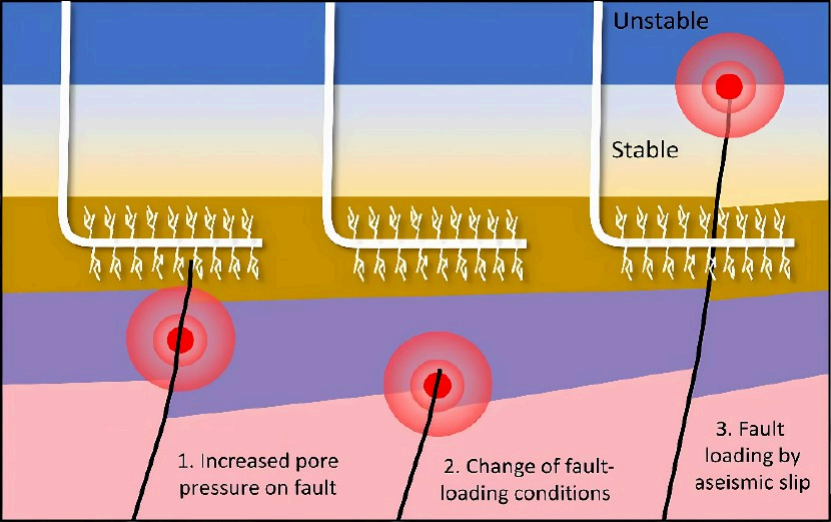
Three potential mechanisms via which fluid injection triggers seismic activity: (1) immediate effect of raising pore pressure on faults; (2) changes in the conditions of fault loading; and (3) aseismic slip-induced seismic slip loading. This figure was reproduced with permission from ref 342. Copyright 2022 Springer Nature.

THMC coupling analysis provides the methodological basis for risk quantification, prediction, and prevention. Snell et al.^336^ discovered that pore pressure greatly shortens the time it takes for an earthquake to start, which makes early warning systems more important. Luu et al.^337^ emphasized the importance of poroelastic stress in simulating seismic activity rates. In THM coupling, the influence of temperature fluctuations on fault frictional stability is particularly significant when the temperature differentials are considerable. Studies on HMC coupling indicate that the mineral composition of fault infill profoundly influences slip modes by modulating velocity-strengthening and velocity-weakening tendencies.^338^ Current research concentrates on the comprehensive integration of multiphase flow, geomechanics, and rate-and-state friction laws to precisely replicate the whole continuum from aseismic slip to seismic nucleation.^339^ In order to control future risks, we need to make high-precision, multifield coupled prediction models that work with real-time microseismic monitoring to combine data and make adjustments as needed. This will create a “simulation-prediction-monitoring-feedback” management closed loop.^340^ Engineering practices can lower the risk of earthquakes to an acceptable level by improving injection strategies (for example, using brine extraction to control pressure), avoiding faults that are under a lot of stress, and making sure that the project is safe for the environment and has the support of the community.^341^

### 4.7 Perspectives and Prospects

There are still some big problems with putting THMC multifield coupling into CGS. Numerical simulation approaches like TOUGH and CMG have a lot of potential for site screening, wellbore integrity evaluation, and storage evolution analysis. However, their prediction accuracy and engineering reliability are still restricted by how complicated the models are and how much it costs to process. Particularly in areas such as long-term reaction kinetics, multiscale heterogeneity characterization, and relative permeability hysteresis effects, existing models still struggle to fully capture the nonlinear feedback mechanisms of THMC processes. Caprock and fault integrity assessments still largely rely on simplified assumptions, lacking dynamic integration of MC degradation and thermal stress evolution, leading to uncertainties in risk assessment. Although the degradation mechanisms of wellbore materials in CO_2_-brine environments have been studied experimentally, upscaling them and integrating them into full-cycle integrity management remains challenging. These issues hinder the effective translation of THMC coupling analysis from mechanistic research to engineering decision-making. Figure 21 systematically summarizes the current challenges and future development directions for THMC coupling applications.

**Figure 21:**
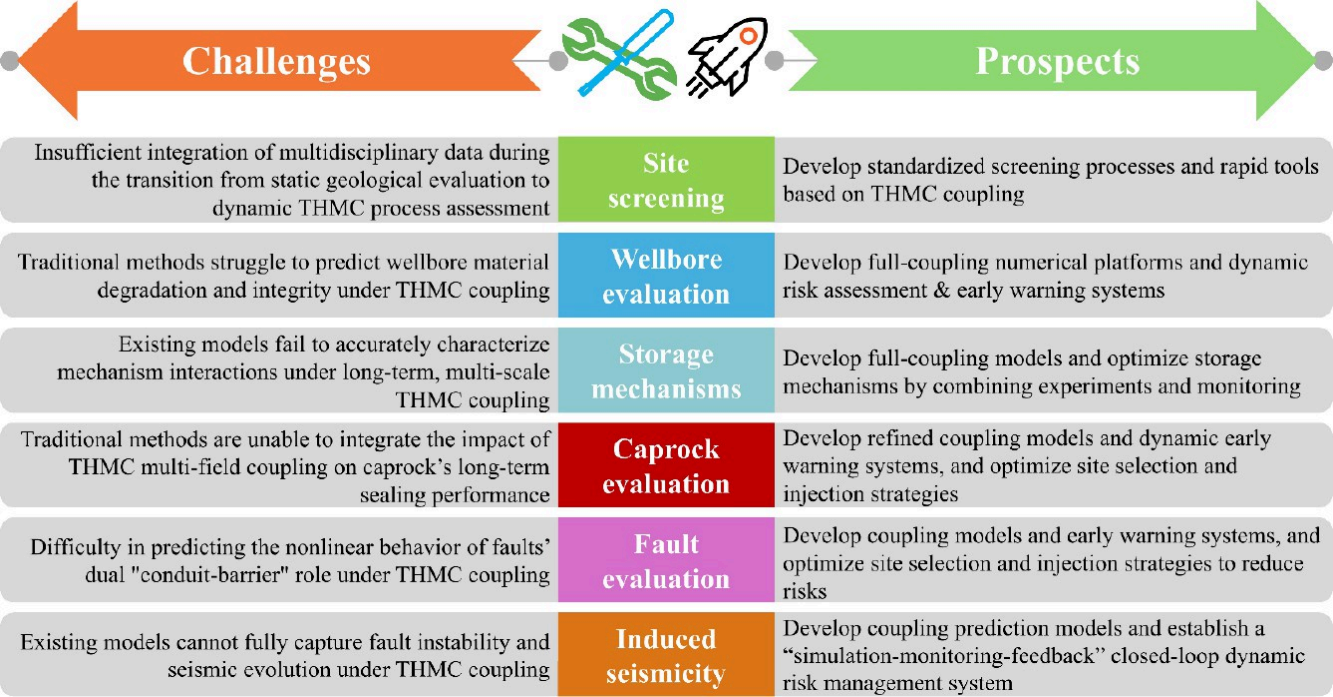
Current challenges and future prospects for THMC coupling applications.

In the future, THMC coupling research will develop toward greater refinement, integration, and intelligence. On one hand, it is necessary to develop fully coupled, multiscale numerical platforms that integrate the dynamic interactions between microscopic material behavior and macroscopic multiphase flow, chemical reactions, and mechanical deformation, to enhance the predictive capability for the long-term evolution of storage systems. On the other hand, real-time data from fiber optic sensing, microseismic monitoring, etc., should be combined with data assimilation techniques to build an integrated “simulation-prediction-early warning” system, enhancing site safety assessment and dynamic risk management capabilities. At the engineering level, the long-term stability and public acceptance of the storage system can be markedly improved by optimizing injection strategies (e.g., regulating temperature, pressure, and rate), utilizing engineered barriers to facilitate the synergistic effects of storage mechanisms, and selectively choosing geological formations with advantageous THMC response attributes. Only through deep integration of interdisciplinary collaboration, model validation, and field practice can THMC coupling analysis effectively support the transition of CGS from the demonstration phase to large-scale commercial application.

## 5 Challenges and Future Prospects

THMC coupling simulation plays an important role in understanding the mechanisms of CGS, assessing risks, and guiding engineering design, but its transition from theory to practice still faces multiple challenges. Figure 22 summarizes the key issues and development directions for current THMC applications. First, computational efficiency and scale coupling are the main current technical bottlenecks.^123^ Although a fully coupled simulation can accurately capture multifield nonlinear feedbacks, its high computational cost limits its application at the field scale and over centennial time scales.^122^ Furthermore, the physical processes span significantly from the pore scale to the reservoir scale, making it challenging to construct multiscale models that both reflect microscopic mechanisms and are suitable for macroscopic predictions, especially in geological bodies with well-developed fractures and strong heterogeneity; the cross-scale transfer mechanisms of fluid flow, chemical reactions, and mechanical deformation remain unclear, leading to high uncertainty in model predictions.^343^

**Figure 22:**
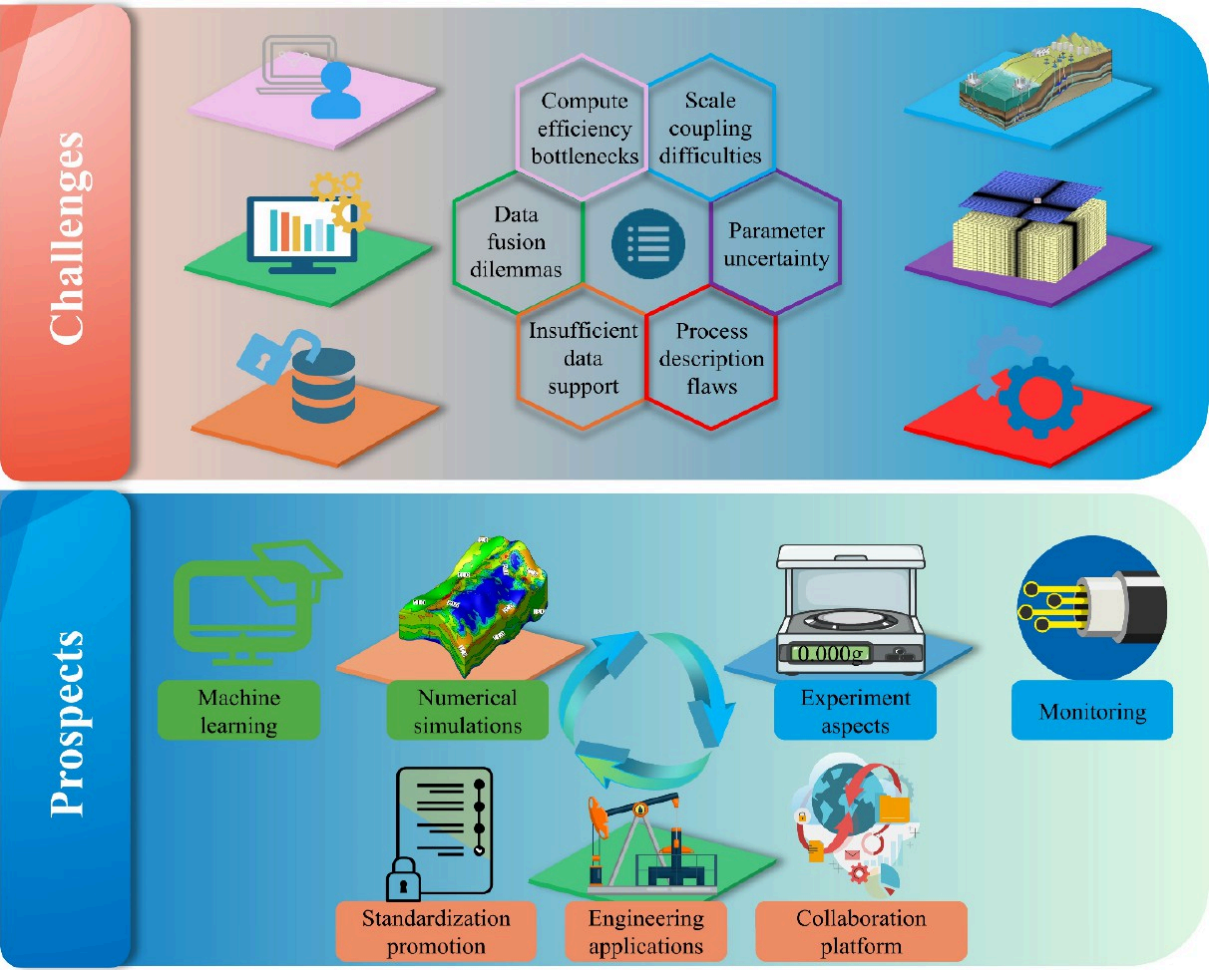
Challenges and future prospects for THMC multiphysics coupling in CGS applications.

Second, uncertainties in model parameters and the depiction of physical processes limit the prediction accuracy and engineering reliability of THMC models. Essential metrics (e.g., in situ permeability, mineral reaction rates, MC coupling coefficients) are challenging to acquire directly at the field scale, frequently depending on laboratory experiments or inversion data, which are plagued by problems of inadequate representativeness and upscaling bias.^344^ In-situ permeability and mineral response rates are critical factors influencing the modeling accuracy. In-situ permeability fluctuates under stress and pore evolution, complicating its precise depiction by laboratory core experiments. The speeds of mineral reactions exhibit significant sensitivity to temperature, pressure, and fluid chemistry, resulting in considerable uncertainties when scaling. Integrating multiscale experiments (pore-core-field) with data assimilation techniques facilitates dynamic parameter inversion and real-time optimization, therefore diminishing uncertainties. Current constitutive relationships predominantly rely on idealized assumptions and inadequately represent the dynamic behavior of actual geological media under THMC pathways; specifically, the impact of the chemical field on mechanical properties is devoid of a cohesive theoretical framework.^326^ Also, things like relative permeability hysteresis and nonequilibrium chemical reactions do not have reliable mathematical representations, which makes the models less accurate for long-term predictions.^345^

THMC models have dual difficulty in validation and data fusion, marked by their “data-hungry” disposition and “validation-deficient” condition. The lack of field examples, including extensive THMC monitoring data, impedes systematic model calibration.^346^ Although laboratory experiments can reveal specific coupling mechanisms, their results are difficult to directly extrapolate to the field scale. The fusion and assimilation of multisource heterogeneous data are still in the exploratory stage, and a standardized data-model collaborative analysis workflow has not yet been formed, limiting the transition of THMC simulation from mechanistic research to engineering decision support.

Looking ahead, the integration of computational methods and intelligent technologies will be key to breaking through the bottlenecks.^11^ An intelligent hybrid coupling framework of “zonal-multiscale-multistrategy” should be developed, employing full coupling in strongly nonlinear regions and sequential or surrogate models in gradually varying regions to balance accuracy and efficiency. Machine learning (ML) methods can bring about a paradigm shift; for instance, constructing high-precision surrogate models can complete simulation tasks that traditionally take days within seconds, greatly accelerating parameter inversion and real-time prediction; data-driven methods also hold promise for discovering previously unrevealed coupling laws from experimental and field data, aiding in the construction of more realistic property models.^347,348^

At the level of model development and experimental validation, it is necessary to promote full-chain innovation “from mechanism to model to monitoring”. Priority should be given to developing constitutive theories that can describe two-way MC feedback, and conducting THMC coupling experiments that simultaneously monitor temperature, pressure, deformation, and chemical composition to provide benchmark validation data for models.^89^ Integrating real-time monitoring technology with data assimilation algorithms, a dynamic feedback system of “simulation-prediction-monitoring-update” should be built to achieve real-time diagnosis of the storage system state and risk early warning, forming an operable digital twin platform.^349^

Finally, from an engineering application perspective, THMC coupling analysis should move toward standardization, modularization, and practicality. Establish model libraries and parameter data sets for different storage scenarios, develop user-friendly integrated simulation platforms, lower the barrier to use, and promote adoption by industry. Through international model comparison projects and the building of open-source communities, we promote model transparency and result reproducibility and construct a cooperative and shared research ecosystem. Only through interdisciplinary collaboration and the deep integration of models and data can THMC coupling analysis support the transition of CGS from demonstration to large-scale safe deployment, providing a scientific basis and technical guarantee for the carbon neutrality goal.

## 6 Summary and Recommendations for Future Work

This paper has systematically reviewed the progress in the application of THMC multifield coupling in CGS. It first elaborated on the fundamental processes of the thermal, hydraulic, mechanical, and chemical fields within the THMC system and their interaction mechanisms, revealing the nonlinear feedback characteristics from dual-field coupling to full coupling. Second, it reviewed mainstream numerical coupling strategies (one-way, sequential, full coupling) and numerical methods (FEM, FVM, FDM, etc.) as well as the applicable scopes of commonly used commercial and open-source software platforms, constructing a comprehensive simulation methodology system. By integrating typical scenarios such as deep saline formations and depleted oil and gas reservoirs, the pivotal role of THMC coupling is explored in site screening, wellbore integrity assessment, quantification of storage mechanisms, caprock and fault stability analysis, and induced seismicity risk assessment. This review aims to furnish researchers and engineers with a thorough knowledge framework, augment the understanding of multiphysics coupling processes in CGS, and lay scientific groundwork for the secure advancement and widespread application of the technology. To solve the basic problems that have already been found, such as low computational efficiency, scale-coupling bottlenecks, parameter uncertainty, and poor data validation, future research needs to be focused on specific problems and make progress through technical methods like intelligent hybrid coupling strategies and multisource data assimilation. This will create a complete logical sequence of “challenge-solution-application implementation.” After a thorough reading of the material, eight suggestions are made:

1. Create advanced hybrid coupling strategies (zonal/adaptive) to provide complete coupling in highly nonlinear areas and sequential/machine learning surrogate models in gradual regions, maximizing accuracy and efficiency.
2. Enhance multiscale (pore-to-field) dynamic property evolution models by sophisticated imaging and in situ testing, improving the modeling of porosity, permeability, and mechanical strength development.
3. Advocate for the integration of multisource data assimilation (fiber optic, microseismic, InSAR) and EnKF-based model validation to establish a dynamic “simulation-monitoring-update” system, therefore diminishing forecast uncertainty.
4. Create a standardized THMC benchmark case library (laboratory-to-field) through collaboration between academics and industry to validate and compare the dependability of simulation methods and codes.
5. Enhance the integration of physics-informed machine learning with physical models for surrogate modeling, finding of constitutive relations, optimization of injection strategies, and detection of THMC precursor signals.
6. Prioritize sophisticated models of caprock and fault long-term evolution (incorporating chemical alteration, fracture dynamics, and fluid migration) to assess self-sealing capability and failure risk for site evaluation.
7. Create user-centric, modular engineering-focused THMC platforms that incorporate geological, flow, mechanical, and chemical modules to reduce access barriers and enhance technology transfer.
8. Enhance multidisciplinary collaboration among geology, geochemistry, rock mechanics, and computational science, as well as facilitate international model comparisons to promote the standardization and maturity of THMC.

## Data Availability

This is a review article. All data discussed are from previously published sources, which are cited in the manuscript.

## Abbreviations

CCUS: Carbon capture, utilization, and storage
CO_2_: Carbon dioxide
CGS: CO_2_ geological storage
IPCC: Intergovernmental Panel on Climate Change
THMC: Thermal-hydraulic-mechanical-chemical
TH: Thermal-hydraulic
HM: Hydraulic-mechanical
HC: Hydraulic-chemical
THM: Thermal-hydraulic-mechanical
HMC: Hydro-chemical-mechanical
THC: Thermo-hydro-chemical
FEM: Finite element method
FVM: Finite volume method
FDM: Finite difference method
DFN: Discrete fracture network
DEM: Discrete element method
CFD: Computational fluid dynamics
EGS: Enhanced geothermal systems
ML: Machine learning
μJT: Joule-Thomson coefficient
